# FCHO controls AP2’s initiating role in endocytosis through a PtdIns(4,5)P_2_-dependent switch

**DOI:** 10.1126/sciadv.abn2018

**Published:** 2022-04-29

**Authors:** Nathan R. Zaccai, Zuzana Kadlecova, Veronica Kane Dickson, Kseniya Korobchevskaya, Jan Kamenicky, Oleksiy Kovtun, Perunthottathu K. Umasankar, Antoni G. Wrobel, Jonathan G. G. Kaufman, Sally R. Gray, Kun Qu, Philip R. Evans, Marco Fritzsche, Filip Sroubek, Stefan Höning, John A. G. Briggs, Bernard T. Kelly, David J. Owen, Linton M. Traub

**Affiliations:** 1 CIMR, University of Cambridge, Biomedical Campus, Hills Road, Cambridge CB2 0XY, UK.; 2 Kennedy Institute of Rheumatology, University of Oxford, Roosevelt Drive, Oxford OX3 7FY, UK.; 3 Czech Academy of Sciences, Institute of Information Theory and Automation, Pod Vodarenskou vezi 4, 182 08 Prague 8, Czech Republic.; 4 MRC LMB Cambridge Biomedical Campus, Cambridge CB2 0QH, UK.; 5 Intracellular Trafficking Laboratory, Transdisciplinary Biology Research Program, Rajiv Gandhi Centre for Biotechnology, Thiruvananthapuram, Kerala, India.; 6 Rosalind Franklin Institute, Harwell Campus, Didcot, UK.; 7 Institute for Biochemistry I, Medical Faculty, University of Cologne, Joseph-Stelzmann-Straße 52, 50931 Cologne, Germany.; 8 Max Planck Institute of Biochemistry, 82152 Martinsried, Germany.; 9 Department of Cell Biology, University of Pittsburgh School of Medicine, 3500 Terrace Street, Pittsburgh, PA, USA.

## Abstract

Clathrin-mediated endocytosis (CME) is the main mechanism by which mammalian cells control their cell surface proteome. Proper operation of the pivotal CME cargo adaptor AP2 requires membrane-localized Fer/Cip4 homology domain-only proteins (FCHO). Here, live-cell enhanced total internal reflection fluorescence–structured illumination microscopy shows that FCHO marks sites of clathrin-coated pit (CCP) initiation, which mature into uniform-sized CCPs comprising a central patch of AP2 and clathrin corralled by an FCHO/Epidermal growth factor potential receptor substrate number 15 (Eps15) ring. We dissect the network of interactions between the FCHO interdomain linker and AP2, which concentrates, orients, tethers, and partially destabilizes closed AP2 at the plasma membrane. AP2’s subsequent membrane deposition drives its opening, which triggers FCHO displacement through steric competition with phosphatidylinositol 4,5-bisphosphate, clathrin, cargo, and CME accessory factors. FCHO can now relocate toward a CCP’s outer edge to engage and activate further AP2s to drive CCP growth/maturation.

## Teaser

FCHO primes AP2 for CCV incorporation, a process that triggers FCHO release to enable activation/recruitment of further AP2s.

## INTRODUCTION

Clathrin-mediated endocytosis (CME) is the chief mechanism for swift and selective uptake of proteins into the intracellular endosomal system of eukaryotes (*1*). It is therefore key to controlling the plasma membrane (PM) proteome and thus cellular life. It is also the system that many invading pathogens, including most viruses, use for cellular entry and establishing infection (*2*, *3*). The clathrin-coated vesicles (CCVs) that mediate CME are formed from clathrin-coated pits (CCPs), which are scattered over the PM and can account for ~2% of the cell membrane surface (*4*, *5*). Clathrin, however, does not contact membranes and their embedded transmembrane protein cargo directly but is attached through membrane-bound clathrin adaptors. The principal endocytic clathrin adaptors are the heterotetrameric AP2 complex and monomeric clathrin assembly lymphoid myeloid leukemia (CALM) (*6*), both of which bind the defining marker of the PM, phosphatidylinositol 4,5-bisphosphate [PtdIns(4,5)P_2_].

**Fig. 1.**
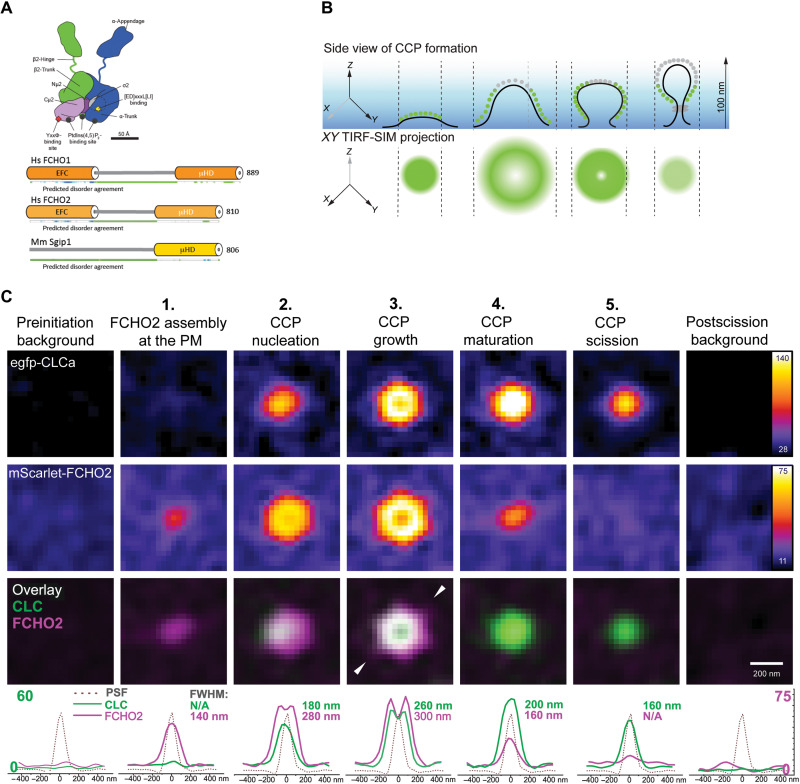
Dual-color live-cell microscopy with high-NA eTIRF-SIM reveals dynamic nanoscopic FCHO2 assembly that predetermines site and size of CCP formation. (**A**) Cartoon representations of mammalian AP2 and muniscin family members FCHO1, FCHO2, and SGIP. AP2 is colored according to subunits as used throughout α (dark blue), β2 (dark green), Nμ2 (dark purple), Cμ2 (pale purple), and σ2 (cyan). Muniscins have FBAR and MHD domains joined by unstructured interdomain linkers. (**B**) Top: Side-view representation of a forming CCP imaged by eTIRF-SIM with an illumination depth of 100 nm (blue gradient). Clathrin is depicted either in green when visible or gray when not visible because of the exponentially decaying evanescent wave of TIRF. Bottom: 2D projection of clathrin (green) in a forming CCP along its axis of invagination into the plane of the PM. (**C**) eTIRF-SIM images of mScarlet-FCHO2^WT^ and egfp-CLCa in different phases of CCP formation. Region of interest (ROI) containing single CCPs in a time series was dissected into phases depending on the appearance of FCHO2 or egfp-CLCa. ROIs with individual CCPs in different phases were assembled into a z-stack and averaged across 150 CCPs in three different cells. The bottom row shows comparison of not normalized intensity distribution profiles (along the line indicated with white triangles) for CLC and FCHO2 (green and magenta, respectively) at different time points together with their full width at half maximum (FWHM). The black dotted line shows the measured point spread function (PSF). N/A, not applicable.

The AP2 complex, composed of α, β2, μ2, and σ2 subunits (Fig. 1A), is pivotal to mammalian CCV formation (*7*), and its deletion is embryonically lethal in mice (*8*). Once recruited onto the cytosolic face of the PM, AP2 coordinates the assembly of a network of 300 to 400 proteins of ~30 identities that comprise an endocytic CCP via an array of dynamic, micromolar *K*_D_ strength protein/protein interactions in a finely choreographed process that takes only 1 to 2 min (*9*). How, why, and where CCPs are triggered to form on the PM are not clear.

Cytosolic AP2 exists in a functionally closed, “locked” conformation that is incapable of simultaneously using its four PtdIns(4,5)P_2_-binding sites to contact the membrane. The binding sites for both of its cognate YxxΦ and [DE]xxxL[LI] cargo sorting signals are also occluded as is its primary LLNLD clathrin-binding motif, and to function in CME, AP2 must be both recruited to the PM and activated, i.e., undergo a large-scale conformational reorientation of its subunits to permit full PtdIns(4,5)P_2_, cargo, and clathrin binding (*10*–*14*). Current models for CCV initiation propose the simultaneous arrival of the three pioneer proteins Eps15, FCHO, and AP2 to form membrane-tethered nanoclusters (*15*–*17*) in which AP2 activation occurs via a molecular mechanism whose details are obscure.

In vertebrates, there are three FCHO paralogs (muniscins): The *FCHO1* gene is expressed primarily in neuronal and lymphoid cells, and *FCHO*2 is ubiquitously produced (*18*–*20*), while *SGIP1* expression is largely restricted to neuronal tissue (*21*). Complete *FCHO2* gene ablation in mice is postpartum lethal. Homozygous recessive *FCHO1* mutants cause immunosuppression and impaired CME, which lead to death if not treated (*22*, *23*). The reported effects on CME of FCHO depletion in cells have varied considerably from a relatively modest inhibition to complete block (*16*, *24*–*28*). FCHO1 and FCHO2 are similar in overall domain arrangement (Fig. 1A)—folded N-terminal acidic phospholipid-binding homodimerization F-BAR domains (*29*) and C-terminal folded μ-homology domains (μHD) (*15*) separated by a ~300-residue, low-complexity intrinsically disordered protein linker, which has been suggested to participate in AP2 activation (*11*, *26*, *27*), although how it does so is undetermined. However, the importance of this linker to FCHO function was demonstrated by transfection of Cos7 cells depleted of FCHO2 by short hairpin–mediated RNA with a linker-deleted version of FCHO2 causing increased inhibition of Transferrin Receptor (TfR) uptake as compared to the untransfected cells (*16*).

Immunolabeled deep-etch electron microscopy (EM) replicas (*15*, *30*) indicated that FCHO is localized mainly at the edges of clathrin-coated structures, which was subsequently confirmed by light microscopy (*16*, *31*). Earlier immuno-EM of ultrathin sections had shown similar patterning at the edges of small discrete clathrin-coated structures for Eps15 (*32*), which binds both FCHO and AP2: All three were characterized as being the earliest-stage recruited CME proteins (*33*) and so were termed pioneers. However, because of the absence of a high-accuracy temporal dimension carried out simultaneously and in combination with superresolution spatial analysis, crucial questions remained unaddressed, which include the following: Does FCHO2 appear before clathrin and AP2? Does FCHO undergo spatial reorganization to the CCP periphery with respect to clathrin and AP2? Furthermore, the distribution of AP2 in CCPs/CCVs remains unclear, as it has been shown to be both present throughout them and at their edges (*31*) but also only on their membrane-distal hemispherical parts (*34*).

Since it is of fundamental importance to understanding CME and thus to the maintenance of the PM proteome, we set out to describe the spatial and temporal interrelationships between the key CME components AP2, FCHO/Eps15, and clathrin using live-cell, high–temporal accuracy, superresolution imaging eTIRF-SIM (enhanced total internal reflection fluorescence–structured illumination microscopy). With this achieved, we then deciphered the mechanisms of interaction with AP2 of four short, evolutionarily conserved blocks of sequence within FCHO linkers. X-ray crystallography and cryo-EM in solution [single-particle analysis (SPA)] and on the membrane (tomography and subtomogram averaging) allowed us to understand how these interactions and their relation to AP2’s PtdIns(4,5)P_2_, cargo, clathrin, and regulatory and accessory factor binding sites direct AP2’s PM deposition, activation, and spatial separation from FCHO during CCP formation. Combining all of these data has allowed us to further develop the model for CCP initiation, growth, and maturation at molecular resolution.

## RESULTS

### Superresolution dual-color live-cell microscopy reveals a critical role for dynamic FCHO2 assemblies in determining the fidelity of CCP formation

To understand how FCHO2 dynamic patterning drives CCP nucleation, we used rapid live-cell multicolor eTIRF-SIM and developed a semiautomated analysis pipeline of time-lapse movies (fig. S1A), enabling us to resolve structures as small as 90 nm simultaneously in all channels (*35*) (Fig. 1B) at an imaging speed of one frame every 100 ms. We imaged the formation of CCPs in a U-2 OS cell line in which FCHO2 knockout was followed by stable reconstitution with near-endogenous levels of mScarlet-FCHO2^WT^ (fig. S1, B to D) and stable expression of enhanced green fluorescent protein (egfp) clathrin light chain a (egfp-CLCa).

On the basis of the unique spatial and temporal patterns of FCHO2 in comparison to those of clathrin, we can identify five distinct CCP lifetime phases (Fig. 1, B and C). In phase 1, we observed that FCHO2 forms a circular patch at the PM, and its local concentration increases. Given that the eTIRF-SIM resolution is limited to 90 nm, we cannot formerly disprove that FCHO2 also forms a ring upon its initial assembly at the PM. However, our interpretation of the shape of the first FCHO2 assemblies is based on the previous work by Stachowiak and colleagues (*36*), which demonstrated that the FCHO2 forms a droplet with liquid-like properties. During phase 2, clathrin recruitment and then CCP nucleation begin. Automated quantitative analysis of conventional TIRF time-lapse movies revealed that 96% of all the dynamic and growing FCHO2 assemblies recruit AP2 and mature into CCPs (fig. S2, A to C). FCHO2 preceded AP2 and clathrin recruitment to the membrane, as an FCHO2 signal was observed in the same pixel location at which a CCP lastly appeared, before clathrin or AP2 signals. In agreement with Fig. 1C, we observed that the FCHO2 signal reached its maximum intensity ~10s earlier than the signals for AP2 and clathrin (fig. S2, B and C). Taking into consideration high axial resolution thanks to the TIRF modality, which provides under 100-nm optical sectioning at the basal plane, we can attribute the earlier appearance of FCHO2 to its accumulation at the PM (Fig. 1, B and C).

The semiautomatic quantitative analysis of superresolved eTIRF-SIM movies (movies S1 to S4) revealed that during phase 2, where clathrin recruitment/CCP nucleation starts, the FCHO2 rapidly transformed from a patch into a ring as its abundance increases. To extract the spatial distributions of FCHO2 and CLCa, we plotted their relative radial intensity profile. The distance between the points where the intensity is half of the maximum is 35% larger for FCHO2 in comparison to CLCa. We interpret this as the FCHO2 patch redistributing to the periphery of the nucleating CCP around the membrane invagination, which would be in line with (*16*, *31*). During phase 3 (Fig. 1, B and C), the FCHO2 ring expands further. The clathrin signal also appears annular, presumably due to the Z-projection of the spheroidal dome it must form (*34*, *37*, *38*) and the exponentially decaying intensity of the incident light within evanescent TIR field.

**Fig. 2.**
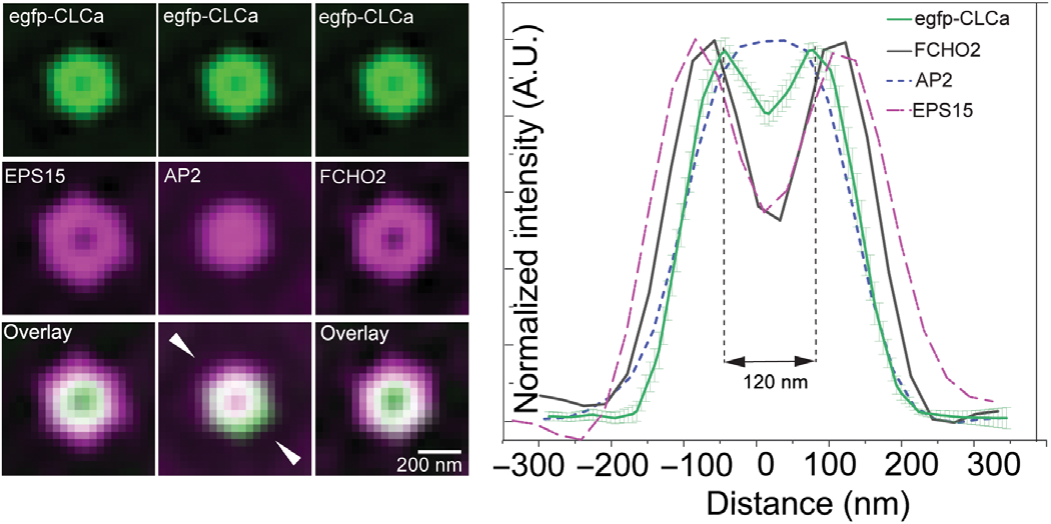
FCHO, Eps15, AP2, and clathrin localization in CCPs. (**Left**) Averaged eTIRF-SIM images of multiple individual ROIs (as in C). The top row (green) shows CLC, and the middle row (magenta) shows pioneer proteins of interest (Eps15, AP2, and FCHO2). The bottom row demonstrates overlay between the channels highlighting the molecule distribution with respect to CLC. mScarlet-FCHO2^WT^ forms an annulus covering the outer rim of the clathrin hemispherical coat of averaged CLC (green line) that is 260 nm. (**Right**) Normalized egfp-CLCa intensity profiles along the line indicated with white triangles of the previous panel. The green solid line shows the CCP intensity profiles obtained from averaging images of 480 CCPs in U-2 OS cells with an estimated FWHM of 260 nm. While AP2 (α-adaptin egfp, shown in blue dashed line) forms a central patch in a mature CCP, Eps15 (polyclonal antibody, magenta dashed line) forms a transition zone with FWHM larger than that of FCHO2 (black) and egfp-CLCa (green). A.U., arbitrary units.

We examined the spatial distribution of AP2, clathrin, FCHO, and Eps15 in the CCP growth phase (phase 3), identified on the basis of an annular reorganization of FCHO in live cells, by immunofluorescence. We found AP2 limited to a central patch, presumably at the distal end of the CCP, in line with (*34*). FCHO2 had been proposed to bind AP2 on the membrane to potentiate its cargo binding (*11*, *15*, *26*). However, Fig. 2 shows that the FCHO segregates from the central AP2 patch into a ring colocalizing with an Eps15 ring [in line with (*5*, *16*, *31*, *32*)], with the FCHO intensity maxima ~50 nm peripheral to the AP2 signal maxima (Fig. 2). This leaves only a thin interface where direct interaction of FCHO2 with open, membrane-attached AP2 would be potentially possible: on the inside of the FCHO ring. A second potential interface exists on the outside of the FCHO ring where FCHO can engage with incoming “cytosolic” closed AP2s. These data suggest that the current proposed model for FCHO function is oversimplified. EPS15 forms a phase-separated “liquid” patch with FCHO2 before CCP nucleation (*36*); however, its function and organization later in CCP lifetime are less well established. Figure 2 shows that Eps15 colocalizes with FCHO2 in the annulus around the central AP2 patch.

We observed that during maturation (phase 4), the FCHO2 signal had already started diminishing and had completely dissipated before scission (phase 5), in line with (*16*) and Merrifield’s early seminal work (*33*). However, since AP2 and FCHO2 have minimal, if any, interaction on the membrane later in a CCP’s lifetime, as they have segregated into separate zones and there are only meager quantities of FCHO2 but large amounts of AP2 in CCVs measured by quantitative mass spectrometry (*6*), they likely leave by different mechanisms: AP2 segregates to the distal end of the CCP, dropping out of the evanescent field rather than leaving the CCP (*39*), whereas FCHO2 must diffuse from the CCP into the PM following competition for PtdIns(4,5)P_2_ by the arrival of late-phase PtdIns(4,5)P_2_-binding endocytic proteins (*40*).

Last, our TIRF data show an inverse correlation between the amount of the early FCHO2 accumulation and the time taken to eventual CCP scission (fig. S2A). This may potentially suggest that a role for FCHO2 is in lowering the energetic and/or kinetic barriers for, i.e., facilitating, CCP formation, presumably through its interaction with AP2 (*11*, *15*, *26*). The data also show that there is a notable size uniformity of all CCPs in both cell lines we tested: The average size of 480 circular CCPs did not deviate by more than 6% from an average diameter of 122 nm in U-2 OS cells and retinal pigment epithelium (RPE) cells (Fig. 2). The calculated surface area is compatible with a resultant lipid vesicle diameter of ~60 nm as is observed for CCVs isolated from cells (*41*).

Emerging from these observations is the crucial mechanistic question of how FCHO drives initial recruitment of AP2 yet subsequently segregates from AP2 (and clathrin), as the CCP grows when its role is meant to be activating AP2. Furthermore, what interactions mediate the dynamic assembly and disassembly of the AP2-FCHO2-PM complex that permits the distinct rearrangements of these components throughout a CCP’s lifetime and how can FCHO facilitate CCP formation/AP2 activation?

### PtdIns(4,5)P_2_ membrane binding drives AP2 opening and competes off FCHO linker

**Fig. 3.**
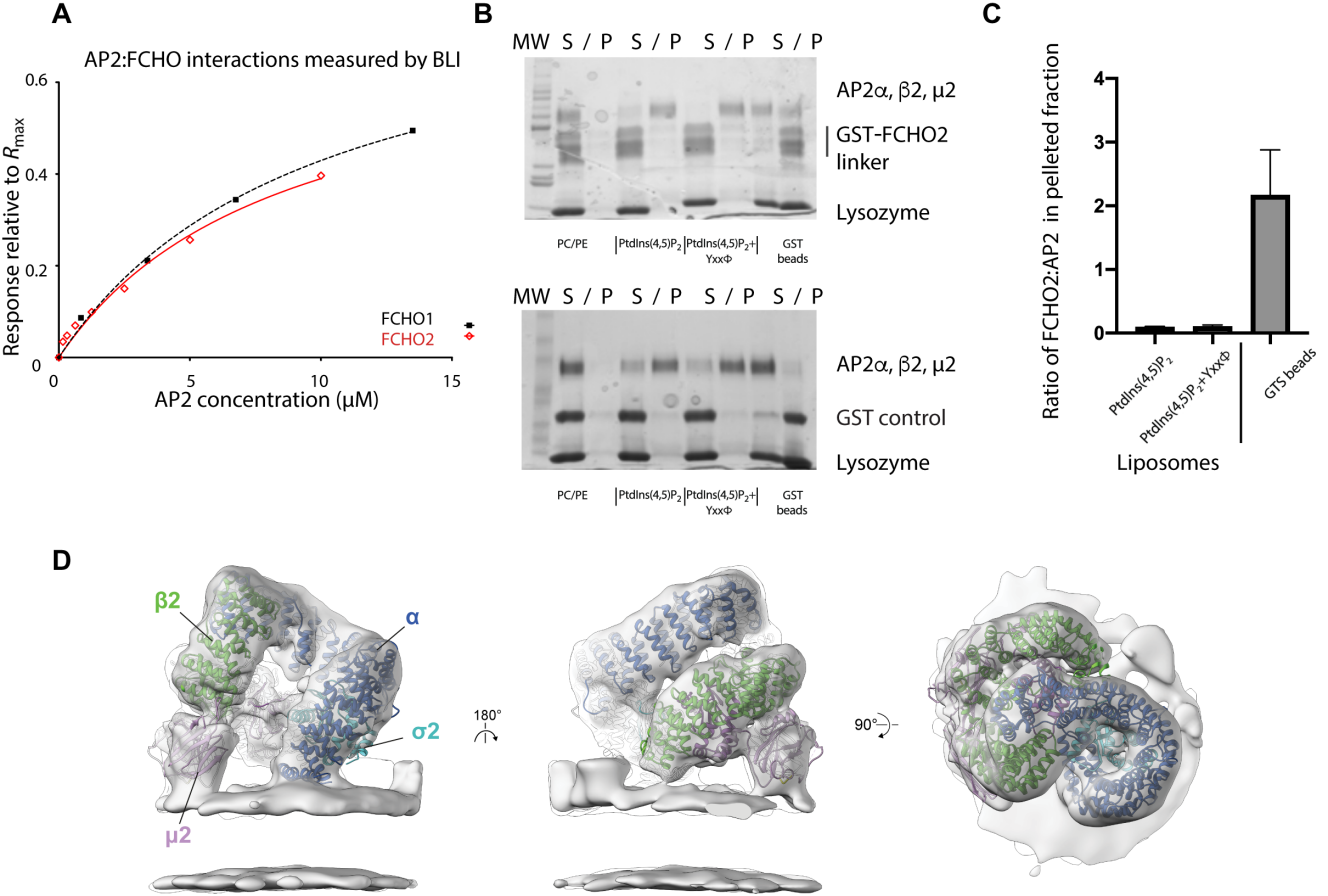
PtdIns(4,5)P_2_-containing membranes compete with FCHO for binding AP2. (**A**) Equilibrium analyses BLI showing similar binding of recombinant AP2 core to FCHO1 linker (residues 316 to 444) with *K*_D_ of 8.5 ± 2.2 μM and to FCHO2 linker (residues 314 to 444) with *K*_D_ of 9.8 ± 1.5 μM. (**B** and **C**) Coomassie-stained SDS–polyacrylamide gel electrophoresis (PAGE) gels of supernatant (S) and pelleted (P) fractions following addition of phosphatidylcholine (PC)/phosphatidylethanolamine (PE), PC/PE/PtdIns(4,5)P_2_, and PC/PE/PtdIns(4,5)P_2_+YxxΦ cargo liposomes to mixtures of glutathione *S*-transferase (GST)-FCHO2 linker (15 μM) and AP2 core (1.25 μM), which glutathione (GSH) Sepharose bead pull-downs show contain GST-FCHO2 linker/AP2 core complex (top) and free GST and AP2 core (bottom). AP2 core binds efficiently to PC/PE/PtdIns(4,5)P_2_ and PC/PE/PtdIns(4,5)P_2_+YxxΦ cargo liposomes, resulting in GST-FCHO2 present in preformed complexes being excluded to the soluble fraction since it contains no membrane binding function. (C) Quantitation of three independent experiments in (B), showing that the ratio of FCHO2:AP2 is ~20-fold higher on beads than on PtdIns(4,5)P_2_-containing liposomes. (**D**) Tomographic structure of AP2 on the membrane in the presence of the FCHO2 linker. The cryo-EM map of AP2 recruited to cargo-free membranes in the presence of the FCHO2 linker, resolved to 9.7 Å, is shown as a gray isosurface. The map is fitted with the previously published ribbon model of AP2 bound to YxxΦ motif–containing membranes in the absence of the FCHO2 linker [Protein Data Bank (PDB) 6YAF]. Here and throughout, subunits are colored: α (dark blue), β2 (green), Nμ2 (dark purple), Cμ2 (pale purple), and σ2 (cyan). Molecular weight (MW).

The unstructured interdomain linkers of FCHO1, FCHO2, and SH3-containing GRB2-like protein 3-interacting protein 1 (sgip1) have been proposed to interact with AP2 (*11*, *16*, *26*, *42*). Biolayer interferometry (BLI) under near-physiological conditions, where AP2 core is known to be in its closed cytosolic conformation (*14*), showed that the *K*_D_ values for the direct interactions between AP2core and FCHO2 linker and between AP2core and FCHO1 linker were ~10 μM (Fig. 3A).

Next, we investigated AP2 membrane recruitment in the presence of the FCHO linker. Addition of PtdIns(4,5)P_2_ and phosphatidylserine (PtdSer) or PtdIns(4,5)P_2_ and PtdSer+YXXØ cargo liposomes to preformed AP2core·FCHO linker complexes in solution results in the AP2 binding to the membranes but causes the FCHO to become displaced from AP2 (in this assay, into the soluble fraction; Fig. 3, B and C). These data indicate that FCHO linkers do not bind to membrane-associated AP2; that is, membrane and FCHO compete for binding to AP2. Nevertheless, FCHO2 linker does cause small but reproducible and significant increases in the steady-state efficiency of AP2 membrane recruitment [~13% for PtdIns(4,5)P_2_-only liposomes and ~22% for liposomes containing both PtdIns(4,5)P_2_ and cargo] (fig. S3A).

Cryo–electron tomography (CET) (Fig. 3D) illustrates that when AP2 is added to liposomes containing PtdIns(4,5)P_2_ and PtdSer but devoid of cargo, AP2 still assumes an open conformation, with PtdIns(4,5)P_2_-binding Basic Region 3 (BR3) and BR4 basic patches on Cμ2 (*43*) and the N termini of α and β2, all simultaneously bound to the membrane surface (fig. S3, B to D). This conformation of AP2 is open and identical to the structure it adopts when cargo is also present in the membrane (*14*). It demonstrates that interaction with high concentrations of free PtdIns(4,5)P_2_/PtdSer alone is sufficient to drive AP2 into an active conformation. When a fivefold molar excess of FCHO linker is also included in the preparation of a cryo–electron tomography sample, the same open conformation of AP2 is obtained, but even at subnanometer resolution, there appears to be no FCHO linker electron density, in line with liposome pull-downs (fig. S3, B to D). These data are supported by the absence of FCHO in mature CCVs (*6*) and our observed segregation of AP2 and FCHO during CCP maturation. Together, these data indicated that FCHO2 binds AP2 in solution (in its closed, inactive conformation) to potentiate AP2 membrane deposition, but the two do not remain bound to each other on the membrane.

### Identifying and dissecting the AP2 binding sequence blocks in FCHO linkers

We set out to understand the molecular mechanism of FCHO·AP2 interaction and to explain why FCHO apparently does not bind AP2 on the membrane, and hence, we see them largely segregate in cells during CCP formation. The ubiquitously expressed FCHO2 (*19*) was used as it is less prone to degradation.

**Fig. 4.**
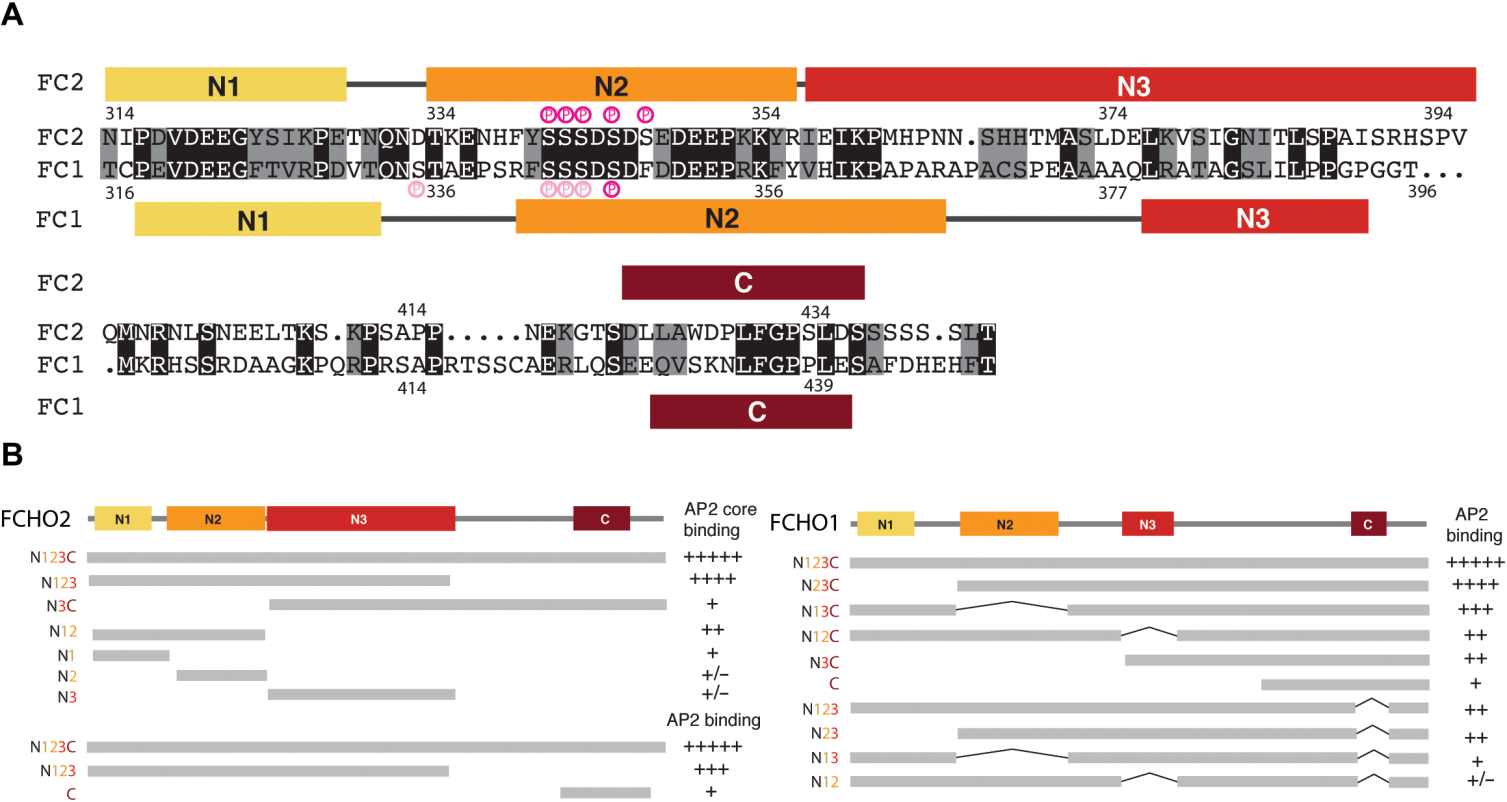
Four sequence blocks in FCHO linkers and the effects of their deletion on AP2 binding. (**A**) Human FCHO2 (FC2 top) and FCHO1 (FC1 bottom) interdomain linker sequences aligned with identities highlighted in black and conservation in gray. Consensus CK2 phosphorylation sites are shown in dark pink and hierarchical CK2 sites in light pink. The conserved sequences blocks N1, N2, N3, and C assessed on the basis of conservations between sequences from across species and in the case of FCHO2, also on the basis of structure, are colored yellow, orange, red, and claret, respectively (coloring maintained throughout all subsequent figures). (**B**) Summary of the binding of the deletion constructs indicated on the unstructured FCHO2 and FCHO1 linkers to recombinant AP2 core and AP2 from brain cytosol as indicated. Gels shown in fig. S3.

Phylogenetic analyses of FCHO linkers identified four blocks of potential functional importance on the basis of conservation of sequence and secondary structure prediction: In FCHO2, these were designated as N1 (314 to 328), N2 (334 to 357), N3 (358 to 397), and C (423 to 438) (Fig. 4A and fig. S3E). Single blocks or pairs of blocks showed little detectable binding to AP2 cores under near-physiological conditions (Fig. 4B and fig. S3F), and removal of C block has little inhibitory effect. Binding of FCHO2 linker to full AP2 from brain cytosol was affected by C block deletion, while isolated C block alone showed some binding, which hinted at the C block being involved in interacting with AP2 appendages (see later). Broadly similar results were obtained with FCHO1 (Fig. 4B and fig. S3, G and H). Moreover, a synergistic (more than additive) association of AP2 with glutathione *S*-transferase (GST)–FCHO1–N1+N2+N3 and GST-FCHO1 C or between GST-FCHO1 (N2+N3) and GST-FCHO1 C occurs when both blocks are coimmobilized together upon glutathione (GSH) Sepharose (Fig. 4B and fig. S3I).

### Closed AP2 binds FCHO2 linker

**Fig. 5.**
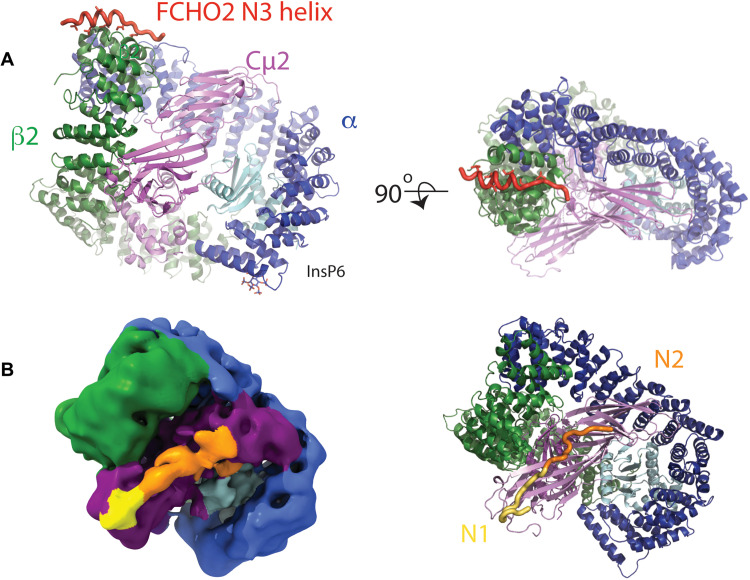
FCHO linker N1, N2, and N3 blocks can bind and activate closed and part-open conformers of AP2. (**A**) Structure of AP2:β2FCHO2linker chimera showing the position of the bound N3 helix (red) and InsP6 in orthogonal as indicated. (**B**) Left: Single-particle EM reconstruction of AP2:μ2FCHO2-N1+N2+N3 chimera highlighting the position of FCHO2-N1+N2 (see N1N2-enriched subclass in fig. S5 and table S6). The map, locally filtered with a global resolution of 7 Å, is colored according to AP2 subunit, the positions of which are derived from a fitted closed AP2 core (PDB 2vgl), and the approximate locations of N1 (yellow) and N2 (orange) are also indicated. N1 is persistent in all reconstructions of AP2:μ2FCHO2-N1+N2+N3 chimera, whereas N2 appears to be less fixed (fig. S5): N3 is not seen in any single-particle reconstructions. The position of N1 is in agreement with x-ray crystallographic structures (Fig. 4). N2 appears to extend from the N1 density associated with the Cμ2 BR3 site across the broad, positively charged BR4 surface patch of Cμ2. Right: Ribbon representation of AP2 in the same orientation as the left panel with FCHO linker shown in worm representation.

We were, however, unable to crystallize AP2 core in the presence of free FCHO2 linker. To increase the effective *K*_D_ of the interaction between FCHO2 linker and AP2 shown by BLI to have a *K*_D_ of ~10 μM and thereby improve the chances of isolating crystals of the complex, a number of chimeras were made consisting of different combinations of blocks attached to different AP2 subunits. Most resulted in protein with poor purification properties and failed to provide material suitable for crystallization. However, one, a chimera of AP2 core with FCHO2 N3-C region fused to the β2 C terminus (AP2:β2FCHO2-N3+C), was crystallized in the presence of the PtdIns(4,5)P_2_ analog d-myo-inositol-1,2,3,4,5,6-hexakisphosphate (InsP6), diffracted to 2.9-Å resolution, and was solved by molecular replacement (MR) (Fig. 3A and fig. S4, A and B) (*44*). AP2 was in its closed, inactive conformation (not unexpected as no YXXØ cargo peptide was present). A predicted helix from the N3 block occupied the hydrophobic groove formed between α helices 26 and 27 of β2-trunk (Fig. 5A and fig. S4B). The interaction is mediated by hydrophobic side chains from FCHO2, the pattern of which and a helical prediction (*45*) are conserved in FCHO1 and in Sgip1 N3 sequences (Fig. 4B and fig. S3E). Extrapolating from the finding that N3 must provide a major contribution to any FCHO·AP2 interaction, we studied the effect of deleting N3 in vivo on CCP phenotype. In line with the structure, while a construct comprising the FBAR and linker of FCHO can rescue the abnormal, dysregulated AP2 assembly CCP phenotype (a decreased number of larger AP2-containing structures and an increased number of tiny abortive structures) seen in 1E HeLa cells (*26*) similarly to wild-type (wt) full-length GFPFCHO, a GFPBAR+linker construct in which N3 is replaced with a similarly sized linker rich in glycine, serine, and alanine residues cannot rescue the phenotype (fig. S4C), presumably due to compromised AP2 binding.

We turned to cryo-EM (SPA) to try to resolve further details of the interaction. A chimera in which N1-N3 of the FCHO2 linker was fused to the C terminus of Cμ2 via a 30-residue linker (AP2:μ2FCHO2-N1+N2+N3) was suitable and was used for SPA structure determination. A His_10_ affinity tag placed at the C terminus of N3 allowed us to ensure that purified material contained full-length N1-N3 linker using a Ni–nitrilotriacetic acid (NTA) agarose recapture step. The resulting chimeric μ2+N1 N2 N3 band was of the correct molecular weight and showed no degradation (fig. S4D). Refined AP2 complex particles (83%) were used to determine a majority class structure at a resolution of 4 Å (table S6): Its conformation was closed (fig. S4, E to G), closely resembling the previously reported closed structure of AP2 core in physiological buffer [Electron Microscopy Data Bank (EMDB) 10747 and Protein Data Bank (PDB) 6yae]. Reconstructions of several conformational subclasses were made (fig. S5): The subclass with the clearest FCHO linker EM density, which was determined using ~^1^/_3_ of the particles used in the 7-Å structure determination, is shown in Fig. 5B. Extra features were clearly present in the EM map near the BR3 PtdIns(4,5)P_2_-binding site on Cμ2 (*43*), as well as also being observed near the BR4 PtdIns(4,5)P_2_-binding site, which we surmised would likely be N1 and N2, respectively (Fig. 5B and fig. S4, E to G). The lower resolution of the N1 and N2 density (fig. S5) suggests some conformational flexibility and/or substoichiometric binding. No EM density for N3 was present in any of our reconstructions.

### Molecular mechanism of FCHO2 linker N-terminus binding to AP2 Cμ2

**Fig. 6.**
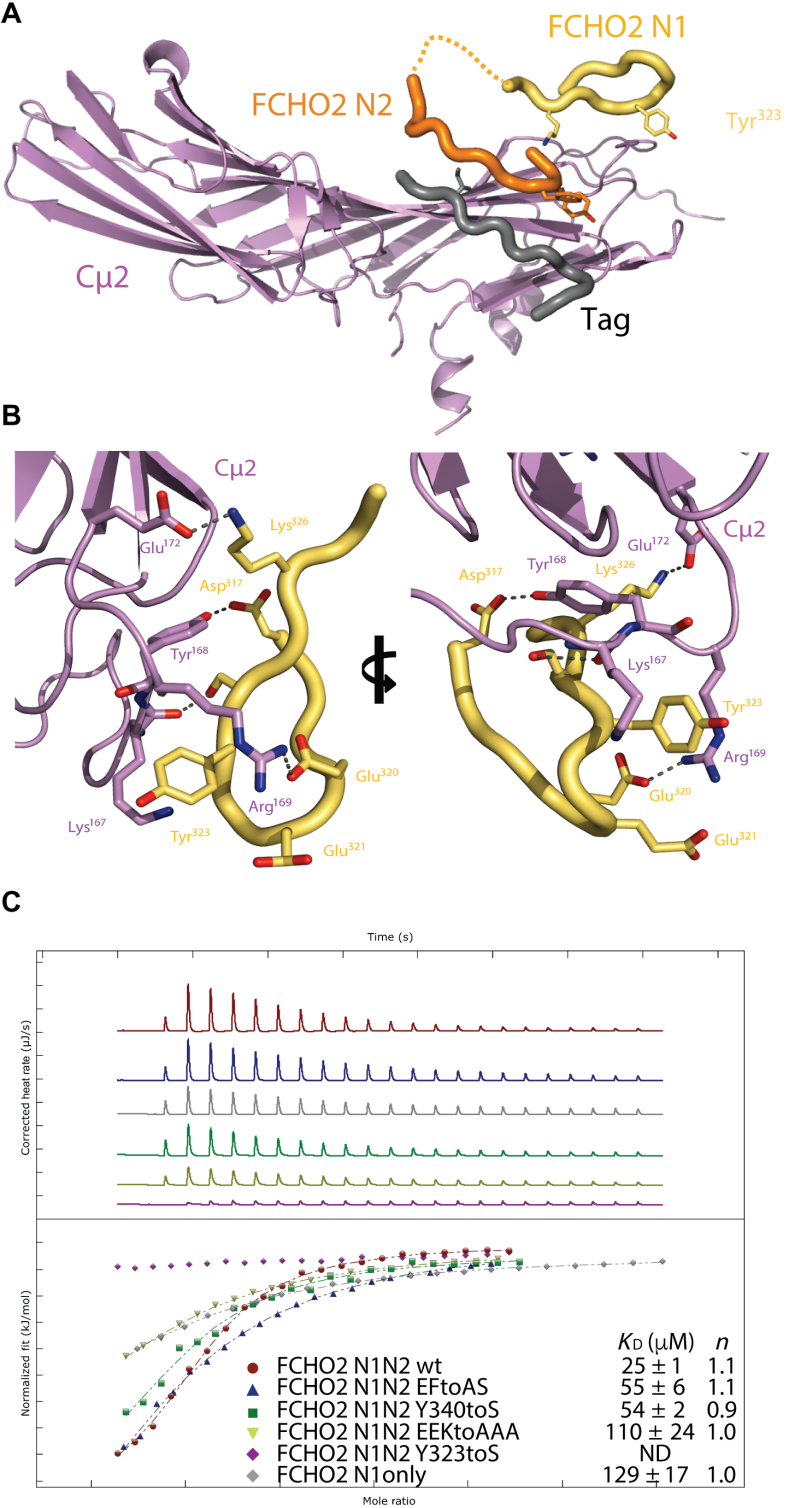
Binding of N1 to Cμ2. Overall positioning (**A**) and molecular details (**B**) of the N1 block bound to isolated Cμ2, allowing better definition of N1’s binding to Cμ2 in whole AP2. The position of N2 and an affinity tag (gray) occupying the YxxΦ-binding site are also shown. The N1 interaction occurs mainly via complementary charged interactions between Asp^318^, Glu^320^, and Glu^321^ and the Cμ2 BR3 PtdIns(4,5)P_2_-binding site containing Lys^167^, Tyr^168^, and Arg^169^. (**C**) Confirmation of N1 binding site on Cμ2 by ITC using structure-directed mutants. E321A+E322A+K326A reduces binding ~5-fold from ~25 μM, and mutating Y323S renders binding unmeasurably weak and not determinable (ND). Mutations that fall outside the binding interface, Y340S and E336A+F339S, have little effect in binding. Deleting N2 also reduces binding fivefold.

To confirm the identity of the extra, Cμ2-proximal electron density as the N terminus of FCHO2 linker, chimeras of Cμ2 alone with N1+N2 attached to the C terminus via 30-residue unstructured synthetic glycine-, serine-, and alanine-rich flexible linkers were then created, crystallized, and their structures solved at resolutions varying from 2.0 to 2.6 Å by MR using an unliganded version of Cμ2 as a search model. Diffracting crystals of (Cμ2-FCHO2-N1+N2) with either a thrombin-cleaved GST tag (Fig. 6A) or an N-terminal His_6_ tag (fig. S6 A) were obtained in the absence of free YXXØ cargo peptide. The Y pocket is variously filled by parts of the tags of an adjacent Cμ2 molecule. Residues from N1 wound around the N terminus of Cμ2 (Fig. 6, A and B, and fig. S6, A and B). Muniscin family conserved side chains (Fig. 4A) make key interactions with Cμ2 residues 167 to 170 (Fig. 6B), which form the BR3 PtdIns(4,5)P_2_-binding site (*12*, *43*). Isothermal titration calorimetry (ITC) measurements (Fig. 6C) showed the binding of FCHO2-N1+N2 to have a *K*_D_ of ~25 μM. Mutation of key binding residues in Cμ2 (E320A+E321A+K326A) reduced binding fivefold, and mutating Tyr^323^ to serine rendered it unmeasurably weak. The FCHO linker does not directly interact with the Y or Ø cargo motif binding pockets in any structure but does pass close by before its main chain changes direction at FCHO2 Pro^327^. The weak density that follows on from N1 adopts a range of conformations forming crystal packing contacts, which we believe to be nonphysiological (mutations FCHO2 Y340S and E336A+F339S have little effect on N1-N2 binding).

### Binding mechanism of poly acidic N2 to BR4 PtdIns(4,5)P_2_-binding site on μ2

**Fig. 7.**
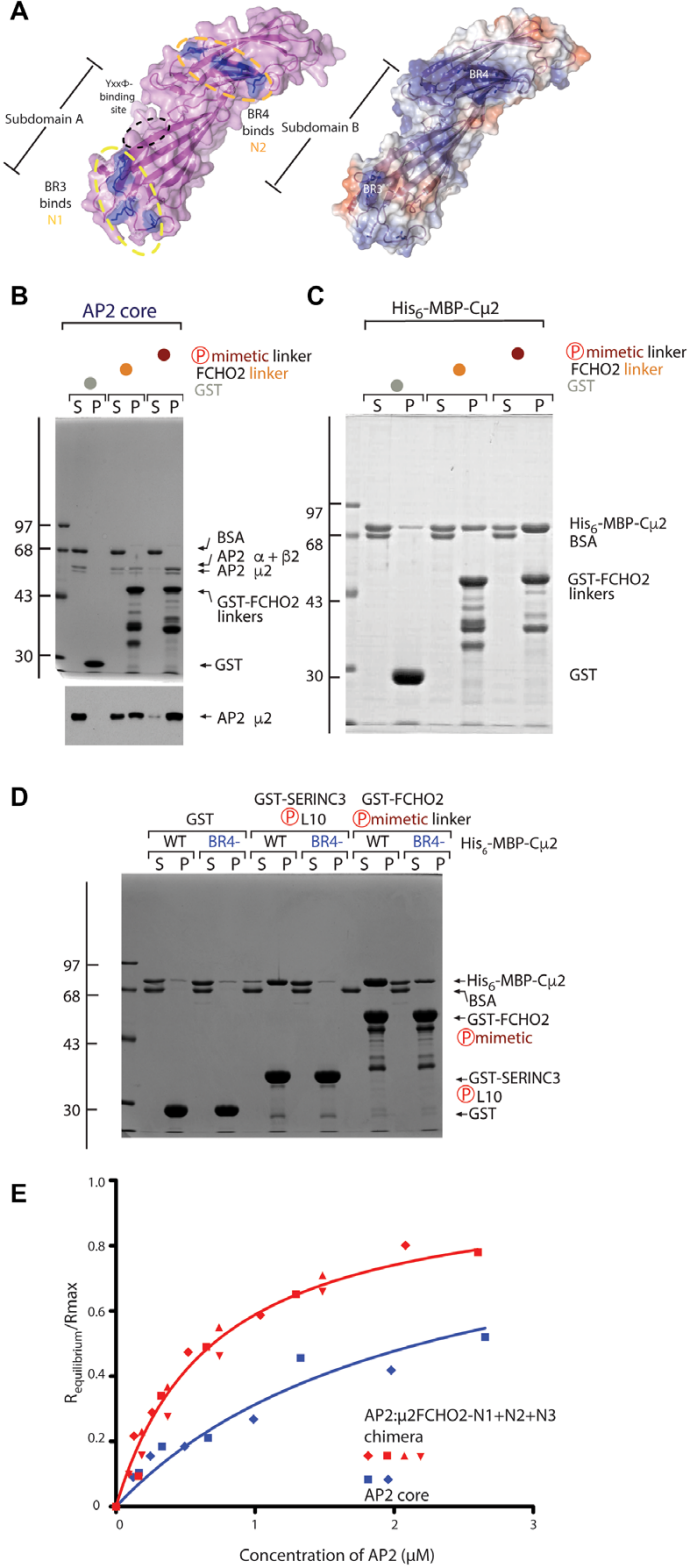
Binding of N2 to Cμ2 and AP2. (**A**) AP2 core binding to GST, GST-FCHO2 WT, and phosphomimetic linker in pellet fraction (P). SDS-PAGE Coomassie-stained (top) and anti-μ2 subunit immunoblot (below). There is a clear loss of AP2core from the supernatant (S) fraction with either increased linker binding to phosphomimetic over FCHO2^WT^ linker in (P). (**B**) Affinity isolation of His_6_-MBP-Cμ2 by GST, GST-FCHO2 WT, and phosphomimetic linker in pellet fraction (P) shown by Coomassie staining of SDS-PAGE. The extent of Cμ2 binding in the pellet (P) fractions is enhanced by S→E phosphomimetic substitution. (**C**) Surface representations of the Cμ2 subdomain structure with positively charged residues from BR3 and BR4 highlighted in dark blue (left) and (right) same view of Cμ2 colored for electrostatic potential contoured from −0.5 V (red) to +0.5 V (blue). (**D**) WT or BR4-mutated (K339A, K341A, K343A, and K345A) His_6_-MBP-Cμ2 binding to GST, GST-SERINC3-L10, or GST-FCHO2 phosphomimetic linker as indicated by Coomassie blue staining of SDS-PAGE. Robust binding of SERINC3-L10 or GST-FCHO2 phosphomimetic linker is shown in (P), which is severely inhibited by the BR4- compound mutation. Note that the binding of GST-FCHO2 phosphomimetic linker is less affected than the binding of SERINC3-L10, confirming the presence of another binding site for the FCHO2 linker on Cμ2, i.e., BR3 patch. (**E**) Binding of GST-YxxΦ to AP2 core (blue) and AP2:μ2FCHO2-N1+N2+N3 chimera (red) in physiological buffer shown by BLI. AP2:β2FCHO2linker chimera shows ~3-fold tighter binding to TGN38 due to faster on and slower off rates than does AP2 core.

Whereas N1+N2 binds Cμ2 with a *K*_D_ of around 25 μM, N1 alone (i.e., deletion of N2) reduces binding by ~5-fold (Fig. 6C), demonstrating that N2 plays a role in binding AP2. The short, acidic N2 segment contains six glutamate/aspartate residues interspersed with serines, which are phosphorylated casein kinase 2 (CK2) sites (Fig. 4A) (*46*). These serine residues were mutated to glutamate to create a phosphomimetic version of the FCHO linker. Figure 7 (A and B) shows that both GST·FCHO2 wild type linker and its phosphomimetic version are able to bind to AP2 and to isolated Cμ2, with the glutamate substitution markedly increasing binding, suggesting that N2 binds to a basic patch. Since the EM density following N1 in the AP2:μ2FCHO2-N1+N2+N3 chimera SPA (Fig. 5B) is located adjacent to the highly positively charged region of Cμ2 that includes Lys^341^, Lys^343^, Lys^345^, and Lys^354^ (*47*) termed the BR4 PtdIns(4,5)P_2_-binding site (Fig. 7C) (*43*), this density can be assigned as N2. In support of this, mutating four of BR4’s basic residues to alanines (BR4-) caused substantial reduction in binding of GST·FCHO2 linker to Cμ2 (Fig. 7D): The lower level of binding reduction of Serinc3 loop results from binding Cμ2 only at BR4 and not to BR3 (*43*), whereas in FCHO2, N1 can still bind to BR3 even when the BR4:N2 interaction is disrupted. These data demonstrate that N1 and N2 can bind through largely charged interactions to the BR3 and BR4 PtdIns(4,5)P_2_-binding sites on Cμ2 when AP2 is in its closed conformation. The binding of N1 to BR3 and N2 to BR4, combined with the fact that cryo-EM tomography shows that BR3 and BR4 sit directly on the membrane, provides the structural explanation for our observation that AP2 cannot simultaneously bind the FCHO2 linker and membrane-localized PtdIns(4,5)P_2_.

### Conformational destabilization in AP2 is favored by binding FCHO2

Compared to our previous analyses of AP2 by SPA EM under identical conditions (*14*), a much higher degree of particle heterogeneity was seen with AP2:μ2FCHO2-N1+N2+N3, likely indicating a decrease in stability of the closed conformation due to the presence of the FCHO linker. Previous crystallographic studies have shown that during the transition of AP2 between closed and open conformations, the μ2 subunit is repositioned, and the gap between the α-solenoid stacks of the α- and β2-trunks is reduced by ~16 Å. In the range of particles observed of AP2:μ2FCHO2-N1+N2+N3, there is a contraction of the gap between the solenoids of ~14 Å (movie S4), yet without the movement of Cμ2 out of the AP2 bowl. A key residue known to latch β2 and Cu2 together in the phi pocket, β2Val365, is no longer occluded in the FCHO-enriched SPA reconstruction of AP2:μ2FCHO2-N1+N2+N3 when compared to AP2 alone, further supporting a destabilization of “closed” AP2 when FCHO2 is bound. Classification of the AP2:μ2FCHO2-N1+N2+N3 particles (see earlier) suggests that a significant minority of the particles adopt a “Cμ2-out” conformation where the Cμ2 subunit is no longer contained within the AP2 bowl (fig. S5) [that there was no degradation of μ2 was confirmed by SDS–polyacrylamide gel electrophoresis (PAGE); fig. S4D]. This conformation is reminiscent of that seen where Cμ2 is deleted and the AP2 bowl relaxes to the open conformation (*13*). Because of the high degree of flexibility, we did not determine a higher-resolution structure of this conformation. In conclusion, significant conformational variability was present in the AP2:μ2FCHO2-N1+N2+N3 chimera as compared to apo AP2 (*14*), with conformations ranging from closed through part open to a fully open bowl connected to a randomly positioned Cμ2.

We have been unable to visualize N1+N2+N3 simultaneously bound to closed AP2. This is despite, first, each block being able to bind individually to closed AP2 and, second, that in a model of such a complex (fig. S7A), the distance FCHO needs to cross to link the Cμ2/N1 and β2/N3 binding sites intramolecularly (~25 Å) is easily spanned by the intervening linker sequence. This could be explained by increased flexibility, conformational heterogeneity, or both. A likely cause of this is that closed AP2 with simultaneously bound N1-N2-N3 is of higher energy than a number of more flexible, part-open AP2 structures similarly bound to N1-N2-N3: Such a range of structures would typically have Cμ2 at least partly displaced from the AP2 bowl but not into a fixed-energy minimum position, and hence, we will now term them “not closed.” Simultaneous binding of N1, N2, and N3 may well also lower the energy barrier between closed and not closed. The net result of both these processes would be to shift the equilibrium of AP2 core in solution away from closed. The energetic equilibrium between closed and open must be finely balanced as indicated by the distribution of particle conformers in SPA and be easy to “tip” as witnessed by the increased protease sensitivity and rescue of FCHO deletion phenotypes in *Caenorhabditis elegans* (*11*) caused by the introduction of any one of many mutations scattered all over AP2. This explanation is also borne out by our observation that all chimeras containing AP2 core attached to a full-length FCHO linker continuously lost material during purification unlike apo AP2 core, suggesting that a destabilizing shift in equilibrium away from closed had occurred in these chimeras.

In solution in 250 mM NaCl, AP2 is closed and hence unable to bind N-terminal fluorescein-labeled YxxΦ TGN38 peptide due to occlusion of its Cμ2-binding site by β2. Inhibition of binding can be relieved by the addition of ~20-mer heparin to represent the highly negatively charged PtdIns(4,5)P_2_-containing PM (*12*). Any conformation of AP2 in which Cμ2 is not tightly abutted against β2-trunk should be capable of binding YxxΦ sequences. BLI (Fig. 7E) indicates that under near-physiological conditions, apo AP2 core must be somewhat destabilized, as it shows some binding to GST-TGN38. In comparison, AP2:β2FCHO2 linker chimera showed ~3-fold tighter binding to GST-TGN38 due to faster on and slower off rates: Note that this is in line with speculations in (*11*, *15*, *26*) but is only relevant to a situation in solution and not in vivo where TGN38 cargo is membrane embedded. The shift in kinetics and thus binding caused by the presence of FCHO2 linker in solution is relatively modest but, when combined with FCHO’s ability to concentrate AP2 at the surface of the PM, would bring AP2 membrane recruitment and cargo binding into the physiologically required time frame.

In considering the mechanism behind destabilization of closed AP2 by the FCHO linker, the interface between Cμ2 and β2 is of particular interest since β2 residues Tyr^405^ and Val^365^ bind directly to Cμ2 at the positions occupied by the “xx” and Φ residues, respectively, of cargo and are key to holding AP2 closed (*10*). The position of FCHO N1 and N2 on Cμ2 and N3 on β2-trunk could function to provide anchor points on a closed AP2. This would position the linker between N1 and N2 such that it would clash with the backbone of the β2 400 to 410 loop, which is part of the β2:Cμ2 interface. It is quite possible that one “not-closed” structure could actually be a conformer that we previously termed open+ (*48*). Using the experimentally derived positions of N1 and N3 α helix binding sites on Cμ2 and β2, a model was created of open+ bound to the FCHO linker (fig. S7B). In this, the distance between anchor points would again be ~25 Å, and FCHO could be easily positioned to contact BR4, while the YxxΦ cargo site would now be free and accessible. To reach the membrane-bound open conformation from “open+,” only a simple translation of 9 Å and a rotation of 90° of Cμ2 with respect to Nμ2 are required. Thus, we propose that FCHO initially interacts with closed AP2 and, in a mechanism likely driven by interference with the β2:Cμ2 interface, AP2 is shifted in its energetic landscape away from the closed conformation, reducing the occlusion of the YxxΦ-binding pocket on Cμ2 and facilitating its rapid opening by the planar PtdIns(4,5)P_2_-containing membrane: This agrees with our observation that FCHO potentiates the efficiency of AP2’s binding to membranes (see earlier).

### Structural analysis of FCHO2 linker binding to open AP2 in solution

**Fig. 8.**
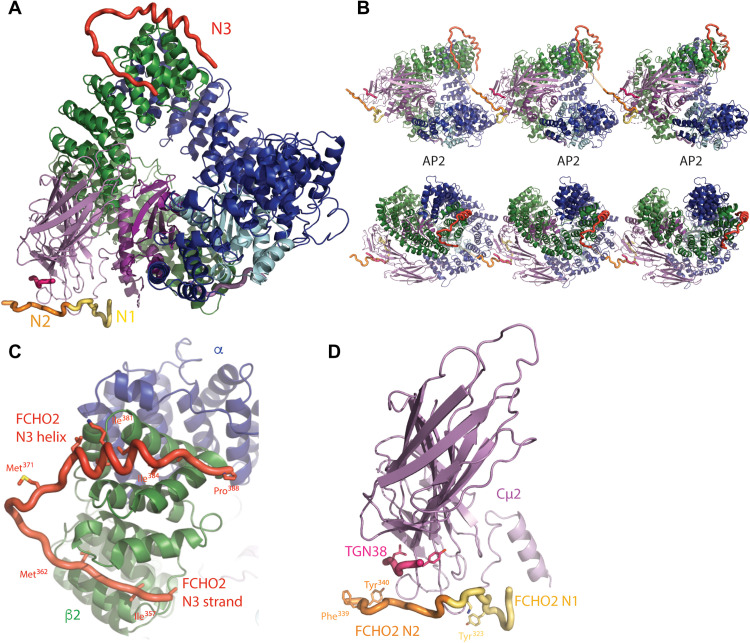
The binding of N1 and N3 FCHO blocks to open AP2. (**A**) Overall structure of a single AP2 core in its open form bound to N1 and N2 from one FCHO linker and N3 from a different FCHO linker (N1 in yellow, N2 in orange, N3 in red, and TGN38 cargo in pink in all subsequent images). (**B**) Orthogonal views of three AP2s in crystals formed in the presence of FCHO. The N1 and N3 blocks of any given linker bind to different AP2 molecules. N1 binds to the N terminus of Cμ2, and N3 binds to the C terminus of β2 from a different AP2. (**C**) Close-up of the binding of N3 helix and preceding strand in hydrophobic troughs between helices 26 and 27 and between 22 and 24, respectively, of β2 in open AP2/FCHO2 linker complex crystals. (**D**) Close-up of the position of the binding site of N1 and N2 to Cμ2 in open AP2/FCHO2 linker complex crystals.

Since FCHO destabilizes the closed conformation of AP2, TGN38 YxxΦ cargo peptide (DYQRLN) was added in the hope of stabilizing an open form of AP2 in complex with FCHO2 linker. The resulting crystals diffracted to a resolution of ~3.3 Å in space group *P*2_1_. The structure was solved by MR (*44*) using the bowl of the open AP2 and Cμ2 as sequential search models and demonstrated that AP2 was in the open conformation (Fig. 8, A and B). In addition, to clear electron density for Cμ2-bound YXXØ cargo and for the σ2-bound pseudo-dileucine cargo myc tag (*12*), there was additional electron density for N3 assuming a helix of ~3 turns occupying the same β2-trunk groove formed between α helices 26 and 27 (Fig. 8, A and C, and fig. S7C). In addition, there was a short unstructured segment of peptide chain contiguous with it packing in the groove between helices 22 and 24. With the assistance of anomalous diffraction collected from crystals in which the FCHO2 linker was selenomethionine-labeled, we were able to fully assign all of the N3 FCHO2 linker block, confirming our earlier identification of the N3 binding site on closed AP2.

Transposing the position of N1 when bound to isolated Cμ2 onto this open AP2 core:FCHO2 linker complex structure demonstrates that the final piece of additional electron density is N1 (Fig. 8, A and D, and fig. S7D) and that it binds in the same way in all the structures described in this work. The weaker electron density following N1, which “points” at the start of an N3 attached to the β2 subunit of an adjacent AP2 molecule in the crystal, is the start of N2. Hence, the FCHO linkers appear to cross-link adjacent AP2s and thus provide a major crystal lattice packing interaction (Fig. 8B). These data both confirm the locations of the N1 and N3 binding sites and suggest that if N1 and N3 are simultaneously bound as anchor points, then the closed conformer becomes destabilized. However, since our earlier data also show that binding of AP2 to membrane fully opens the AP2 and simultaneously outcompetes off the FCHO linker, i.e., FCHO and hence membrane binding cannot be cotemporal, this solution structure cannot be physiologically relevant. While the N1 and N3 binding sites are unobstructed in AP2’s open conformation, the distance (~75 Å) that needs to be spanned is on the very edge of what is theoretically possible if all of the intervening sequence was completely extended; in practice, this will not occur since even glycine-serine peptide chains only exist in a state that is considerably less than “fully extended” (*49*). These observations, along with the absence of FCHO cross-linked and arrayed AP2s in cryo-EM tomography and SPA, lend further support to the conclusion that FCHO does not bind membrane-attached open AP2.

### Molecular mechanisms of FCHO2 linker’s C block binding to AP2’s α-appendage and Cμ2

The final highly conserved region of the FCHO linkers is block C, which contains an LFGPXL sequence (Fig. 4A and fig. S3E) separated from N3 by a variable-length (30- to 40-residue) disordered linker. Since FCHO1 and FCHO2 have been suggested to bind the AP2 appendages (*27*), we investigated this possibility.

**Fig. 9.**
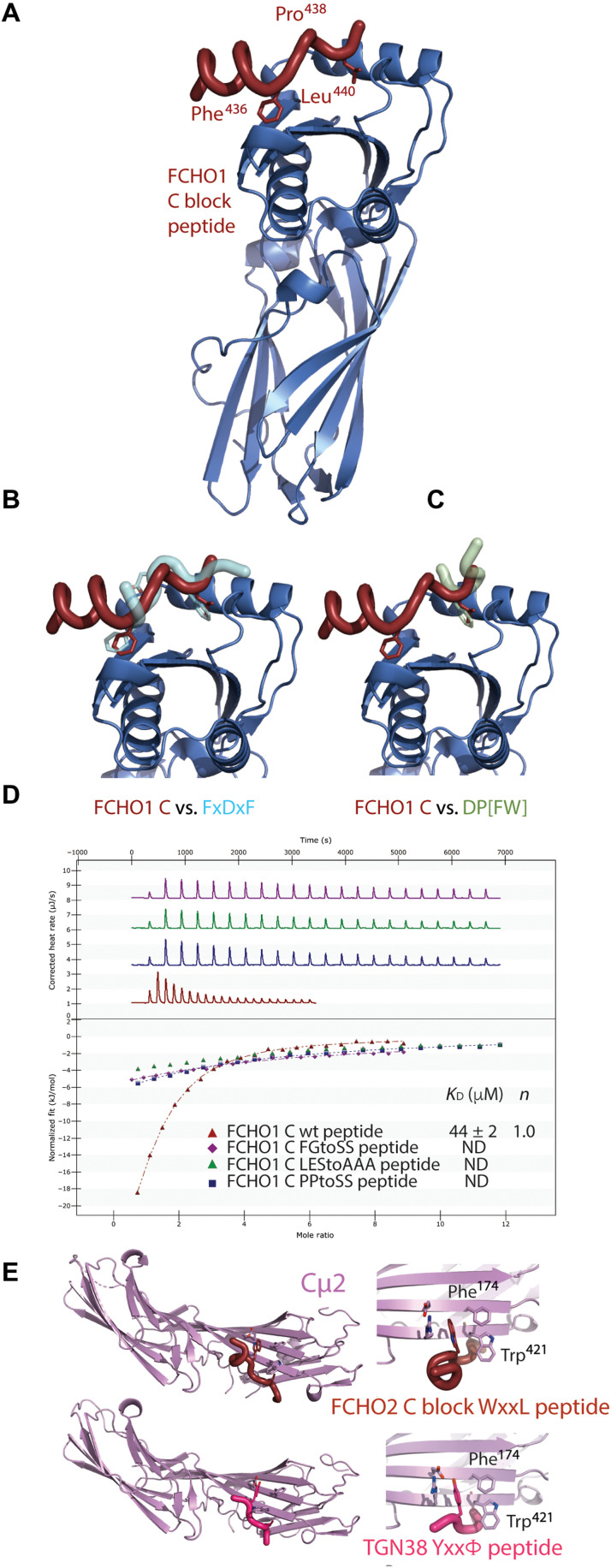
Alternative binding sites of the FCHO linker C block on α-appendage and Cμ2. (**A**) Structure of α-appendage (blue) showing the FCHO1 linker C block (LFGPPLES) bound to the platform subdomain at 1.4-Å resolution. Key binding residues are indicated. The residues preceding the core FGPPL motif for a short helix that packs against the side of the platform subdomain. (**B** and **C**) Superposition of FCHO1 linker C block with (left) bound FxDxF peptide (PDB 1KYU7) and (right) bound DPF peptide (PDB 1KYU), both shown in semitransparent representation. The FCHO1 SKNLFGPPLES peptide binds in the opposite orientation to FxDxF and DPF peptides whose final F and central F, respectively, sit in the same hydrophobic pocket as the C-terminal L of LFGPPL. (**D**) ITC showing ~50 μM *K*_D_ binding of LFGPPL to α-appendage. Mutation of FG to SS, LES to AAA, and PP to SS all reduces binding to weaker than 500 μM *K*_D_. (**E**) Top: 1.7-Å resolution structure of FCHO2 WxxL motif of the WDPLFGPSLDS C block (claret) bound to Cμ2 at its YxxΦ motif–binding site (*K*_D_ of 56 μM and electron density; see fig. S6) in perpendicular views. Bottom: Same perpendicular views of Cμ2 bound to TGN38 YxxΦ cargo peptide (pink).

ITC shows that various similar-length control peptides containing several hydrophobic residues do not bind α-appendage, whereas the C block of FCHO1 binds with a *K*_D_ of ~50 μM, while the FCHO2 and SGIP C blocks bind with a *K*_D_ of 100 to 200 μM (fig. S8A). No binding was observed between the different C blocks and the AP2 β2-appendage. Crystals of the α-appendage were grown in space group *C*222_1_ in the presence of a 10-fold molar excess of FCHO1 C peptide (residues 426 to 448, SEEQVSKNLFGPPLESAFDHEH), which diffracted to 1.4-Å resolution. The structure was solved by MR using an unliganded α-appendage structure (PDB 3hs8) as the search model. Density for the peptide was clearly visible and could be unambiguously built (Fig. 9A and fig. S8B). Although the peptide bound to the top platform site of α-appendage in the same position as FXDXF and DP[FW] motifs (*50*–*52*), the orientation is opposite to that in these previously determined structures (Fig. 9, B and C). The conserved hydrophobic interactions by the FCHO1 LFGPXL Phe^436^ and Leu^440^ (equivalent to FCHO2 residues 431 and 435) anchored the peptide to the α-appendage in the same positions as the phenylalanines of the FXDXF motif, facilitated, in part, by FCHO1 Pro^438^ being in a cis conformation: Mutations in FCHO1 FG to AA, LES to AAA, and PP to GG reduce binding to α-appendage to below detectable levels (Fig. 9D). H bonds between the LFGPXL motif’s main chain and α-appendage side chains further stabilize the interaction. These observations suggest that muniscin LFGPxL ligands could be competed from the α-appendage during the later stages of CCP formation as the number and variety of accessory and regulatory proteins containing FxDxF and DP[FW] motifs increase (*33*)

While FCHO2 clearly shows binding to α-appendage (fig. S6A), we also noticed the presence of a highly conserved W preceding FCHO2’s LFGPxL by two residues. When combined with the first conserved L of the LFGPxL, this results in the creation of a WxxL sequence, which has been proposed as an alternative Cμ2-binding cargo sorting motif (*53*). An FCHO2 peptide incorporating this WxxL bound to Cμ2 in ITC with a *K*_D_ of ~25 μM (fig. S8C), which is of similar strength of binding to many characterized YxxΦ cargo motifs (*54*). Binding was dependent on the presence of the tryptophan side chain as the homologous sequence from FCHO1, which does not contain a tryptophan, showed no binding. Crystals of a complex of Cμ2 and a peptide corresponding to this WxxL signal were grown, diffracted to 1.7-Å resolution, and were solved by MR using 1.9-Å unliganded Cμ2 as a search model. The backbone of the WxxL superimposes with that of a YxxΦ peptide with the tryptophan side chain packing in the Y pocket and the leucine sitting in the Φ pocket (Fig. 9E and fig. S8D). These data suggest the possibility that the C block of FCHO2 has two possible modes of interaction with AP2: one with α-appendage and one with Cμ2. The mode favored will likely be context dependent, and it will likely be affected by multiple spatial and temporal factors in a nascent CCP including how many other α-appendage binding CCV proteins are present and whether the YxxΦ site of Cμ2 is accessible and/or occupied by cargo. These binding modes are likely to be in dynamic equilibrium with each other. The absence of WxxL in the C block of neuronally enriched FCHO1 but its presence in the ubiquitously expressed FCHO2 may reflect the paucity of YxxΦ-containing cargo found in synaptic CCVs (*55*) that could compete out WxxL from Cμ2, which, if it did not occur, could slow or stall CCV formation. Thus, C blocks of all muniscin family members can bind to the α-appendage platform in a manner that is competitive with the binding of FxDxF and DP[FW] motifs of accessory CME proteins, and in addition, the partially overlapping WxxL sequence found only in FCHO2 can bind to the Cμ2 YxxΦ cargo–binding site and therefore potentially stabilize the unclosed but not-yet-open form of AP2.

### The absence of the identified blocks in FCHO alters CCP formation dynamics

Together, these data suggest that the FCHO linker plays an important role in controlling AP2 during CCP formation. The AP2:FCHO interaction has a low micromolar apparent *K*_D_, but each of the four FCHO blocks’ individual interactions has only weak *K*_D_ values, i.e., it works by avidity effects. This results in a highly dynamic, readily reversible, and easily regulatable system. However, it also means that point mutation(s) in any one FCHO block will very likely only have minimal effects on the overall AP2:FCHO interaction. Complete deletion of an entire individual block has only minimal effects on FCHO’s AP2 binding in vitro (Fig. 4, A and B). Although replacing N3 with an equal-length, unrelated sequence alters AP2 puncta morphology as in (*15*, *26*) in vivo (fig. S4C), to see reliable effects on overall CCP behavior, and taking into account the redundant nature of interactions within the CCP component networks (*56*), we decided to investigate the effects of replacing the whole 140-residue FCHO linker with an equally unstructured, flexible random sequence (FCHO2^STRING^) on CCP dynamics. Figure S9A shows a threefold increase in the density of subthreshold initiating clathrin structures in cells with FCHO2 deletion and 30% increase for FCHO2 linker mutant in comparison to the FCHO2^WT^; subthreshold clathrin structures are defined by their failure to yield a productive CCP initiation because a stable CCP nucleus fails to form (*16*). We presume that when a functional FCHO linker is not present, AP2 frequently engages in unsuccessful, transient stochastic interactions with the PM, which are not stabilized by binding cargo and clathrin: This is in line with a model in which FCHO2 reduces energetic and/or kinetic barrier for stable anchoring and concentrating of AP2 at PM.

## DISCUSSION

Formation of a CCP is based on a functional redundancy of its building components. As for any other biological process, however, most of these redundant interactions are important because they have distinct efficiencies under different conditions, and their loss causes an acute reduction in efficiency, which is almost immediately recovered via compensation. Therefore, only perturbations in important nodes of the network, such as those in FCHO2 linker, will lead to a reproducible and measurable phenotype, which is demonstrated in fig. S9A.

**Fig. 10.**
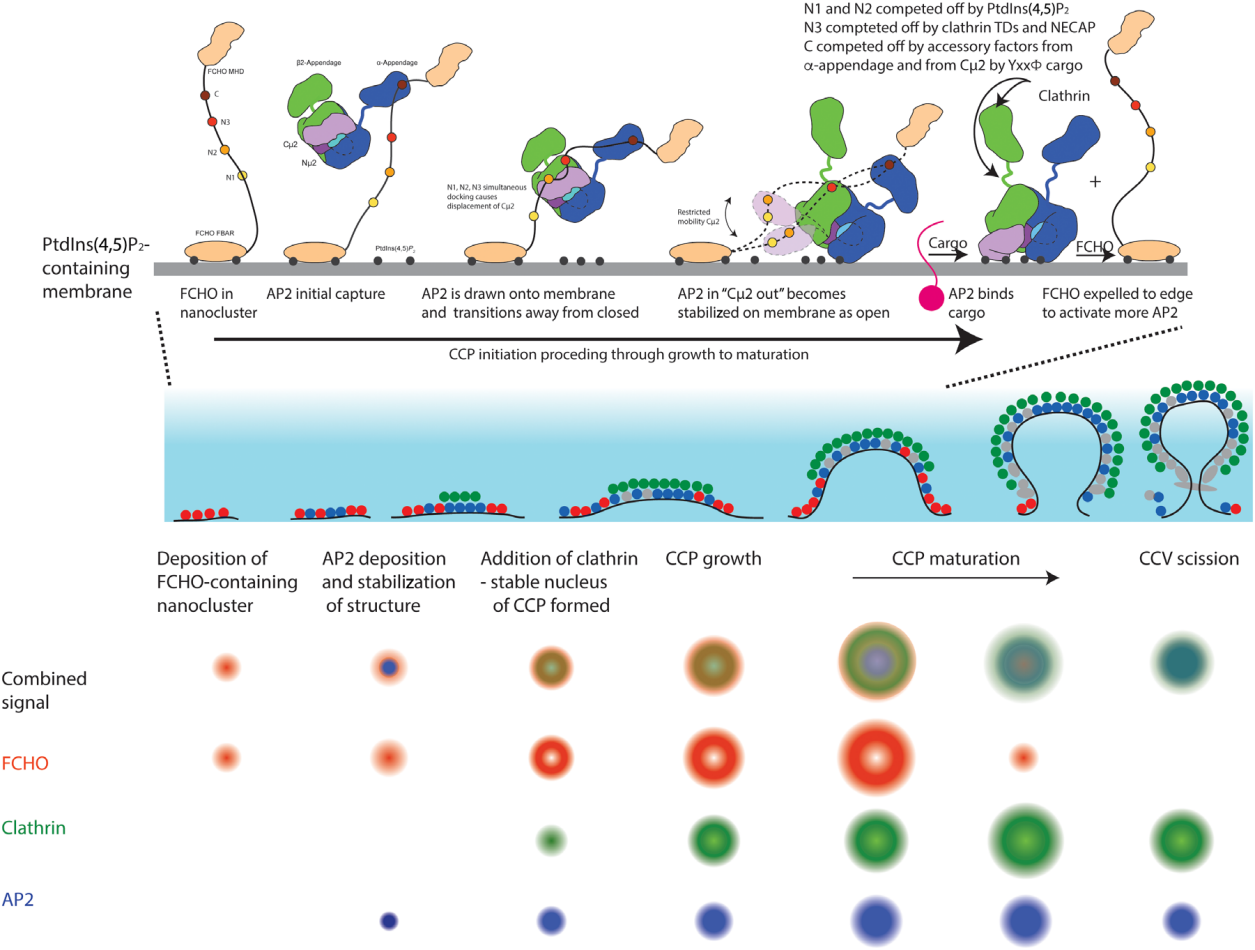
A molecular mechanistic model for the steps in the FCHO-dependent activation of AP2 during CME. (**Top**) FCHO2 bound to the membrane uses its four linker blocks C, N3, N2, and N1 to engage with a closed, cytosolic AP2. Full binding results in the AP2 being orientated and concentrated on the membrane and destabilized to a “μ-out,” flexible conformation. This conformer quickly binds to the membrane, causing it to fully open and N1 and N2 to dissociate. Cargo binding stabilizes this open form, while the binding of polymerized clathrin and regulatory/accessory proteins competes out N3 and C, and the FCHO is expelled to the edge of the forming CCP where it can recruit further AP2s. This process occurs during the phases of CCP growth indicated. TDs, clathrin terminal domains. (**Middle**) Schematic representation perpendicular to the PM of the phases of a CCP’s life from initiation to scission. Left to right: FCHO2 in red; AP2 in blue; clathrin in green; other clathrin adaptors and accessory protein in gray. The pale blue gradient indicates an ~100-nm evanescent field as used for visualization in C. (**Bottom**) Schematic representation of projections of a CCP life cycle’s phases measured or inferred from eTIRF-SIM data. Top line, overlay of respective signals of FCHO2 (red), clathrin (green), and AP2 (blue).

This work demonstrates how and why FCHOs underpin CCV formation. Nanoclusters of FCHOs linked by Eps15 (*15*) into phase-separated microstructures (*36*) bind, concentrate, orientate, and activate AP2 at the PM, potentiating AP2’s productive deposition on the PM to form the nucleus of a CCP. The multiple, dynamic interactions of short sequence blocks within the FCHO interdomain linker with various AP2 subunits ensure that the process is not only of high fidelity and is sufficiently rapid but is also readily reversible to allow proofreading. Since association of AP2 with the membrane triggers the disassembly of AP2·FCHO complexes, this allows reuse of the same FCHO to activate further AP2s (i.e., FCHO could be considered effectively catalytic) to grow a CCP nucleus and, in so doing, provides both temporal processivity and spatial control to CCP formation. Thus, the making and subsequent breaking of the network of FCHO/AP2 interactions are key to the efficient, controlled genesis of endocytic CCVs and thus to the PM proteome. We can now describe an integrated mechanistic model for the initiation of CCP/CCV formation in vivo (Fig. 10) in which the actual mechanism of AP2 opening, driven by PtdIns(4,5)P_2_ binding and stabilized by cargo binding, will be essentially the same in vivo and in vitro [described in (*12*, *13*)] but is facilitated by interaction with FCHO.

### Interaction of AP2 with FCHO orients AP2 with respect to PM and promotes AP2 opening

Of the four FCHO linker blocks, N1 is nearest to the PM as it follows directly after the FCHO FBAR domain, with N2, N3, and C blocks being increasingly distant from the PM. As C can sample the largest volume to find AP2s, it seems likely that C block engagement will be the most frequent founder interaction. Although the order of engagement of subsequent linker blocks can be variable, i.e., a fuzzy process, the most likely subsequent order would be N3, N2, and N1 as this would correctly orientate, “draw down,” and hold an AP2 at the membrane surface, resulting in an AP2·FCHO complex structure resembling that modeled in fig. S9B. Simultaneously, destabilization of AP2’s β2/μ2 interface [possibly enhanced by interactions of α and β2 with membrane PtdIns(4,5)P_2_] shifts the equilibrium of the AP2 away from fully closed. This increases the occupancy of “μ2 out”/not-closed conformations, in which the AP2 bowl has relaxed to a lower-energy, more open-like state [see fig. S5 and (*12*)]. Cμ2 can now sample its local environment on its flexible μ2 interdomain tether so that it can dock onto PtdIns(4,5)P_2_ via its BR3 and BR4 sites and become fully open. AP2’s subsequent local search dimensionality for cargo is effectively reduced from three-dimensional (3D) to 2D: Any cargo will be swiftly bound, further stabilizing an AP2’s open conformation and extending its PM dwell time. When stably open, AP2’s clathrin-binding β2-hinge is released (*13*), and so, clathrin recruitment and polymerization can start: The process is enhanced by virtue of FCHO/Eps15 annular assemblages likely corralling open AP2, with which it will have little or no interaction, to increase its effective concentration and facilitate its cross-linking by a forming clathrin lattice. If sufficient AP2s become stably PM attached through clathrin cross-linking and cargo binding, then the nucleus of a CCV has been formed.

### Interaction of AP2 with membrane and clathrin competes off FCHO interactions

Steric clashing causes competitive binding of PtdIns(4,5)P_2_ and FCHO N1/N2 blocks to Cμ2 BR3 and BR4 sites. This results in N1 and N2 being displaced as AP2 assumes a fully open conformation on the membrane and an FCHO·AP2 complex being significantly weakened as it can be mediated only by N3 interacting with β2-trunk and the C block with α-appendage or Cμ2. C binding will be subject to competition from incoming regulatory and accessory CCV proteins and other clathrin adaptors if it is bound to α-appendage; on the other hand, if it is bound to Cμ2, thus preventing AP2 reclosure and also Cμ2 binding to YxxΦ motifs on cytosolic proteins, it will be expelled by YxxΦ cargo binding. The N3 binding region on β2-trunk overlaps with the clathrin terminal domain layer in assembled clathrin coats [see fig. S9A and (*14*)], which will likely serve to drive N3 off AP2. A further source of competition may also exist for N3 binding to β2-trunk. A very similar sequence to the N3 helix is present in mammalian NECAP “Ex” segment, with the key binding residues being conserved (see consensus sequence between FCHO2 and Necap1 [LI]K[VL][SC]IGNIT) (fig. S9B), along with the prediction of helicity (*45*). Consequently, we believe that N3 and Necap Ex domain can bind in the same place on β2, which could explain the unassigned density seen in SPA structures of μ2Thr^156^-phosphorylated AP2 bound to NECAP (*57*); see fig. S10 (B to D).

### The FCHO-driven concentration, orientation, and activation of AP2 need to be readily reversible

Since CCV formation is energetically expensive, being able to reverse it, i.e., abort CCV formation early (<10 s) when optimal conditions or requirements for a CCV’s formation [e.g., PtdIns(4,5)P_2_ and cargo] are not met (*16*, *28*, *58*), is important to prevent waste of resources. In in vitro reconstructions without time constraints, at high concentrations and without any competition for PtdIns(4,5)P_2_ and cargo (*13*, *14*), stochastic membrane encounter is sufficient in the absence of FCHO to form CCPs/CCVs. In vivo FCHO absence would be expected to cause a high number of abortive events, with CCPs rapidly disassembled. This is observed in cultured cells when FCHO has been deleted [see fig. S8A and (*26*)]. However, only a comparatively modest defect results (on average, 40 to 50% reduction in transferrin internalization rates) (*16*, *24*–*28*), presumably, in part, due to redundancy built into the system, e.g., Eps15/R can still bind AP2 [reviewed in (*59*)]. This reduction in CME rate and the resulting energy expenditure can, it seems, be largely tolerated under the energy-rich conditions in cell culture. In whole organisms, although abortive CCV initiation events do naturally occur (*60*), phenotypes of FCHO deletion are marked and penetrative (*11*, *61*). Last, since N1 and N2, which are involved in the PtdIns(4,5)P_2_-actuated switch on Cμ2, are subject to phosphorylation/dephosphorylation events, e.g., by CK2, the system can receive physiological inputs to finely tune the rate of CME and/or the decision as to whether or not to abort or proceed to ordered completion to the needs and condition of the cell.

### Possible roles for the FCHO/Eps15 corral

Since open AP2 will have coalesced into the center of the forming CCP under the polymerized clathrin and demonstrates only minimal interaction with open and membrane- and cargo-bound AP2, FCHO (cross-linked by Eps15) will be excluded from the center of the forming CCP and displaced to the periphery of the CCP. Thus, FCHO/Eps15 will form a ring. If this phase-separated structure works similarly to the center of a nuclear pore (*62*), allowing only proteins that can bind its constituents to cross it [i.e., most CCP components can bind FCHO (*26*, *27*, *63*) and/or Eps15 (*64*)], then the FCHO/Eps15 ring will bind closed AP2 that is loosely membrane associated on its outside while effectively corralling open, fully PtdIns(4,5)P_2_-bound, clathrin–cross-linked AP2 and to prevent its “escape.” Furthermore, any free phosphoinositides and cargo released by clathrin adaptors within the corral would be inhibited from diffusing out of it, i.e., it acts as a diffusion barrier. As the number of FCHOs and Eps15 in a CCP plateaus early on in its formation, conversion of the FCHO/Eps15 patch into an annulus of increasing radius but decreasing thickness will only be possible up until the point when it is stretched to its maximum length without breaking, i.e., a ring of FCHO dimers linked to each other by Eps15s (*15*). It would then resist further expansion, and the resulting maximum diameter of the annulus should help define the maximum size of a CCP/CCV by the number of clathrin adaptors it can corral inside it, providing an explanation for why all CCPs in the RPE and U-2 OS cells are basically of the same size/intensity. It is therefore possible that the amount of FCHO/Eps15 may play a role in defining the standard CCV size/curvature along with the amount of CALM (*38*, *65*). An annular structure has also been seen in the endocytic CCPs of yeast (*66*), despite its endocytosis being largely actin dependent and so mechanistically different, suggesting that not only this design feature is conserved throughout eukaryotes but also its formation may be a common mechanism in many transport vesicle genesis events.

To conclude, our eTIRF-SIM shows that a small number of FCHO molecules, presumably clustered on the PM by virtue of their FBAR domains binding PtdIns(4,5)P_2_ and PS and by forming phase-separated nanoclusters with Eps15 [Fig. 2, (*15*, *36*)], act as potential sites for CCP nucleation. FCHO achieves this primarily by engaging, recruiting, and activating AP2 at four sites scattered across AP2’s surface. The apparent low micromolar *K*_D_ overall interaction strength of the FCHO·AP2 complex makes AP2 membrane dwell time, concentration, and kinetics commensurate with the physiologically relevant time scales for CME (30 to 90 s) (*9*, *33*, *59*, *67*, *68*). However, by being constructed from multiple weak interactions, the formation of FCHO·AP2 complexes is rapidly reversible. As the CCP grows and matures, the FCHO/Eps15 transforms from a patch into a corralling ring surrounding a central, likely domed patch of membrane-attached AP2 and other clathrin adaptors surmounted by a lattice of clathrin itself. However, since the FCHO·AP2 interaction should only occur at their transition zone/interface and stable membrane attachment of AP2 disrupts FCHO·AP2 complexes, and since AP2s in the central patch will be clustered under a clathrin lattice, released FCHOs will be pushed to the periphery where they can entrap, activate, and transfer further AP2s into the central patch as CCP formation progresses.

## MATERIALS AND METHODS

### Constructs used in this study

For PMIB6mScarlet-3xFlagGGGG-FCHO2^WT^ and PMIB6mScarlet-3xFlagGGGG-FCHO2^STRING^ mutant, residues 314 to 444 were replaced by GNANGNAGAPAGANGNANPGNAGNGAGAPAGANGNANPGNAGNGANAGNGPGNGANAGANAGNANGNAGAGNANGNANPGAGNGAGNANGNANPGAGNGAGNAGNAGANAGNGPGNGANAGANA.

AP2 α-egfp retroviral construct was a gift of S. Schmid (*28*).

pEGFP FCHO FL (residues 1 to 810) and deletion mutants thereof were as follows: pEGFP FCHO BAR only (residues 1 to 275), pEGFP FCHO BAR+linker (residues 1 to 454), and pEGFP FCHO BAR+linkerΔN3 (residues 1 to 365 + GNANGNAGAPAGANGNANPGNAGNGAGAPAGANGNANPGNAGNGANAGNGPGNGANA + 414 to 454).

AP2 core was made with pMWHis_6_β2trunk+mycμ2, pMWGSTαtrunk+σ2.

AP2:μ2FCHO2-N1+N2+N3 chimeric AP2 core was made with pMWGSTαtrunk+σ2 and pMWHis_6_β2trunk+mycμ2-linker-FCHO2 (residues 314 to 444) (linker sequence, GASGSAGSAGPSGAGSAGSAGPSAGSAGSAGSGSAGSAPG).

AP2:β2FCHO2linker was made with pMWGSTαtrunk+σ2 and pMWHis_6_β2trunk (residues 1 to 542)–FCHO2 (residues 358 to 444)+mycμ2.

pMWHis_6_μ2 (residues 160 to 435) wt.

pMWHis_6_μ2 (residues 160 to 435)–linker–FCHO2 (residues 314 to 351) (linker sequence, GASGSAGSAGPSGAGSAGSAGPSAGSAGSA).

pGEXμ2FCHO2 (residues 160 to 435)–linker–FCHO2 (residues 314 to 351) (linker sequence, GASGSAGSAGPSGAGSAGSAGPSAGSAGSAGSGSAGSAPG).

pETHis_6_σ2α2 with σ2 (residues 1 to 142) and α2 (residues 9 to 395) (*69*).

His_6_MBPμ2(residues 160 to 435) wt and BR4 mutant (K339A/K341A/K343A/K345A).

pGEXFCHO2linker wt (residues 314 to 444) and deletion mutants thereof were as follows: pGEXFCHO2 N1-N2 (residues 314 to 356), pGEXFCHO2 N3-C (residues 354 to 444), pGEXFCHO2N1 (residues 314 to 340), pGEXFCHO2N2 (residues 334 to 356), pGEXFCHO2 N3 (residues 354 to 397), and pGEXFCHO2 C (residues 413 to 444).

pGEXFCHO2linkerN1-N3 wt (residues 314 to 394) and mutants thereof of EEK (E320S/E321S/K326S), YS1(Y323S), and YS2(Y340S) EFAS (E336A/F339S).

pGEXFCHO2 phosphomimetic linker (residues 314 to 444) with mutations (S341E, S342E, S343E, S345E, and S347E).

pGEXFCHO1linker wt (N123C) (residues 316 to 444) and deletion mutants thereof were as follows: pGEXFCHO1N23C (residues 334 to 444), pGEXFCHO1N13C (residues 316 to 353 and 354 to 444), pGEXFCHO1N12C (residues 316 to 354 and 414 to 444), pGEXFCHO1N123 (residues 316 to 413), pGEXFCHO1N3C (residues 354 to 444), and pGEXFCHO1C (residues 414 to 444).

pGEX4T2α-appendage (residues 695 to 938).

GST SERINC-L10 (residues 354 to 404) was a gift of R. Singh.

pGEX ARRS control (residues 627 to 635 of βarrestin2).

pGEX4T2 linker (linker sequence, GSGEEVNGDATAGSIPGSTSALLDLSGLDLPPAGTTYPAMPTRPGEQASPEQPSASVSLLDDELMSLGLSDPTPPSGPSLD).

pGEX4T2 linker TGN38 (linker sequence, GSGEEVNGDATAGSIPGSTSALLDLSGLDLPPAGTTYPAMPTRPGEQASPEQPSASVSLLDDELMSLGLSDPTPPSGPSLDYQRLN).

### Antibodies used

Antibodies used in this study were as follows: affinity-purified rabbit polyclonal anti-FCHO1 antibody 1 (1:2500; Traub Laboratory); affinity-purified rabbit polyclonal anti-FCHO2 (1:2500; Traub Laboratory) and NOVUS Biological polyclonal anti-FCHO2 NBP2-32694; affinity-purified rabbit polyclonal anti-AP1/2 β1/β2 subunit GD/1 (1:2500; Traub Laboratory); affinity-purified rabbit polyclonal anti-Eps15 (1:1000; HPA008451, Atlas Antibodies); monoclonal antibody (mAb) directed against the AP2 α subunit clone 8/adaptin α (1:1000; 610502, BD Transduction Laboratories, San Jose, CA); mAb directed against AP2 μ2 subunit clone 31/AP50 (1:500; 611350, BD Transduction Laboratories, San Jose, CA); mAb AP.6 directed against AP2 α subunit, gift of F. Brodsky. Secondary antibodies used were as follows: for blots, donkey anti-rabbit (1:5000; NA934V) or anti-mouse (1:5000; NA931V) horseradish peroxidase conjugates from GE Healthcare Life Sciences (Pittsburgh, PA); for immunofluorescence, goat anti-mouse conjugated to either Alexa Fluor 488 or Alexa Fluor 568 (Invitrogen).

### Cell biology

#### Cell culture

Parental U-2 OS cell line was obtained from the American Type Culture Collection. All cell lines were grown under 5% CO_2_ at 37°C in Dulbecco’s modified Eagle’s medium (DMEM) high-glucose GlutaMAX medium (Thermo Fisher Scientific) supplemented with 10% (v/v) fetal bovine serum (HyClone). The mycoplasma test was performed routinely with the MycoAlert PLUS Mycoplasma Detection Kit (Lonza) to rule out mycoplasma contamination.

#### Generation of FCHO2 knockout U-2 OS cell lines by CRISPR-Cas9n

Genome editing of U-2 OS cells to generate isogenic knockout clones of endogenous FCHO2 (FCHO2-KO) was performed by Genscript. The plasmid containing the human codon-optimized SpCas9 gene with 2A-EGFP and the backbone of single-guide RNA (sgRNA) (*70*) was obtained from Addgene. sgRNA CCTGGAGTATGCCTCCTCTA was designed using the CRISPR-Cas9 target online predictor (*71*) to disrupt exon 2 of FCHO2 (NM_138782.3). The Cas9 plasmid was transfected in the U-2 OS cells, and the transfected and FACS (fluorescence-activated cell sorting)–sorted egfp-positive cells were plated in 96-well plates by limit dilution to generate isogenic single clones. The clones were expanded and screened by Sanger DNA sequencing to identify positive isogenic single knockout clones. Three positive knockout clones (#21, #33, and #31) were further validated by reverse transcription polymerase chain reaction and Western blotting using NOVUS Biological polyclonal anti-FCHO2 NBP2-32694. A wt isogenic clone (#6) was maintained as a negative control to generate isogenic pairs of wt and mutant cell lines.

#### Reconstituting FCHO2-KO cell lines with FCHO2-string and FCHO2-wt constructs

The FCHO2-KO cell lines were reconstituted with mScarlet-FCHO2^WT^ or mScarlet-FCHO2^STRING^ using retrovirus-based expression as previously described. The construct termed mScarlet-FCHO2^STRING^ contained a modified linker based on Gly, Asn, Ala, and Pro residues (predicted to have no secondary structure) with an identical length. The different pools of cell lines with mScarlet-FCHO2^WT^ were obtained by FACS into populations of low, medium, and high FCHO2 expression, and cell pools were further analyzed by Western blotting. The population with the FCHO2 expression equivalent to that of wt U-2 OS cell lines was selected for further experiments. Stable U-2 OS-FCHO2 cell lines carrying fluorescently tagged CLCa were generated as previously described.

AP2-eGFP cells lines were generated by transducing U-2 OS-FCHO2 cells with the corresponding retrovirus as described (*72*). After 72 hours, cells were isolated in populations with different eGFP expression levels by FACS (FACS Flow Cytometry, CIMR). Cell pools with an overexpression of eGFP-tagged AP2 subunits at 2× that of endogenous subunit and at least 70% incorporation were then selected for live-cell imaging experiments.

### Microscopy

#### Live cell TIRF microscopy

Experiments were performed as previously described. Briefly, high-precision #1.5 round coverslips were acid-washed and coated with gelatin (0.2 mg/ml; #2850-22, Corning). All TIRF imaging was carried out with a Zeiss 100× 1.49-NA (numerical aperture) apo TIRF objective, which was mounted on an Elyra PS1 inverted microscope with a Definite Focus System. Time-lapse series were acquired at a frame rate of 0.5 Hz using a PCO Edge 5.5 sCMOS camera. During imaging, cells were maintained at 37°C in DMEM supplemented with 10% fetal bovine serum.

#### TIRF data analysis

Single-channel and dual-channel TIRF movies were analyzed with CME analysis suite. Initiation densities of CCPs and short-lived, subthreshold clathrin structures were determined as previously described (*68*, *73*).

#### Dual-color TIRF data analysis

TIRF microscopy movies of each double-labeled cell line expressing either FCHO2-WT or FCHO2-string were obtained. Master/Slave analysis was used to determine the degree of colocalization in bona fide CCPs throughout their lifetimes. In the first analysis, AP2-egfp or egfp-CLCa was used as a primary channel to determine the evolution of FCHO2 intensity before initiation of bona fide CCP. The number of buffer frames was changed from a default value of 5 to 25 frames. This allowed visualizing background and initial FCHO2 intensities in the same pixel before AP2 or egfp-CLCa appearance. This step effectively excluded any CCPs occurring at hotspots from the analysis: A hotspot is defined as a formation of one CCP followed right after by another. Hence, these CCPs cannot be used for determining the initial de novo recruitment of FCHO2. Inflection time points of AP2 and FCHO2 averaged intensity cohorts were calculated with an approximating cubic polynomial. For complementary analytical approach, we assigned primary channel to FCHO2 dynamic structures to determine their capacity to recruit AP2-egfp and egfp-CLCa.

#### Enhanced total internal reflection fluorescence–structured illumination microscopy

The eTIRF-SIM was performed using a custom-built system described at the Kennedy Institute at the University of Oxford. The detailed description of setup configuration can be found, as well as the applied experimental and analysis pipelines, in (*74*, *75*). Briefly, the system consists of a 1.49-NA 100× objective lens (Olympus) fitted on an inverted microscope body (IX-83, Olympus) and complemented with two high-speed sCMOS cameras (ORCA-Flash4.0, Hamamatsu). The key element of the setup is ferroelectric spatial light modulator (SLM) for generating structured illumination pattern and the TIRF incidence angles. The SLM switching between different modes at very high repetition rates allows fast imaging with up to 200-ms frame rate. To capture egfp-CLCa and FCHO2 dynamics in live-cell eTIRF-SIM movies, 488- and 560-nm wavelengths were used for the excitation. TIRF angles were adjusted for both wavelengths to ensure 100- to 150-nm penetration depth in the axial dimension, which created a well-confined optical sectioning at the basal plane. Nine raw images (three angles and three phases of structured illumination pattern) for each time point and wavelength were acquired to obtain a superresolved image with about twice the resolution of the standard TIRF image. The raw images were processed and reconstructed by a previously described algorithm (*76*). The identical Wiener filter parameter of 0.05 was used for reconstruction of all the images.

### eTIRF-SIM image analysis

#### Preprocessing

Before image analysis, images were chromatically corrected using Multistackreg macro in Fiji (*77*). To ensure high-quality correction, the multicolor images of 100-nm Tetraspeck microsphere (T7279, Thermo Fisher Scientific) images were taken before each dataset and used as transformation matrixes.

#### Automatic detection and tracking

We adopted CMEanalysis software (*68*, *73*), which is an excellent tool for detecting and tracking CCPs in TIRF time-lapse movies. However, in its original version, it is not suitable for reconstructed eTIRF-SIM images with annular CCPs, since it models the fluorescent signal of a CCP as a 2D Gaussian approximation of the microscope point spread function (PSF) above a spatially varying local background. Hence, we averaged the nine raw images acquired for each time point and each channel to generate a single snapshot per channel and time point for CCP detection and tracking in CME analysis with default parameters. The resulting detected spatial coordinates of valid tracks were superimposed on the final eTIRF-SIM reconstructed image. This approximate location and track for each CCP were then manually processed, and each region of interest (ROI) was scrutinized, and manual CCP centroids were repositioned automatically in both channels if necessary. For this purpose, we developed a simple tool with an intuitive GUI to speed up this step. Only single CCPs with canonical lifetime profile were adjusted and used for downstream analysis.

#### Manual postprocessing and intensity analysis, averaging, and bleaching correction

We then manually refined the position of the detection with respect to CCP centroids in eTIRF-SIM data, since the tracks obtained with CME analysis from raw TIRF images only approximated it.

#### Bleaching correction

When extracting individual CCP regions from reconstructed eTIRF-SIM data, we need to compensate for intensity bleaching. We do this by averaging intensities in individual time frames and then fit an exponential curve of the form *Y* = a*exp(b*x) + c*exp.(d*x) to these data, which gives us the final bleaching compensation coefficients.

#### Intensity adjustment and averaging

We preserve original intensities in individual channels. When comparing different channels, we estimate a multiplicative coefficient, which minimizes the difference between average histograms of the channels as a least squares fit. Last, at least 50 snapshots defined as 21 × 21–pixel ROI for a given phase of individual CCPs were assembled in a stack and averaged using Z-projection and intensity averaging in ImageJ.

### Protein expression and purification

Recombinant proteins were expressed in BL21 plyS *Escherichia coli* grown in 2TY media. After OD_600_ (optical density at 600 nm) > 0.6 cell density was reached at 37°C, expression was induced with 0.2 mM isopropyl-β-d-thiogalactopyranoside overnight, shaking at 22°C. Cells were lysed using a cell disruptor (Constant Systems). AP2 core and AP2 chimeras were made in 250 mM NaCl, 10 mM tris (pH 8.7), and 2 mM dithiothreitol (DTT) according to previous protocols (*10*). Briefly, the complexes were initially isolated with GST beads and cleaved overnight with thrombin. The resultant protein was then isolated on Ni-NTA beads, washed with buffer supplemented with 10 mM imidazole, and eluted from the beads with the same buffer supplemented with 300 mM imidazole. After size exclusion chromatography on a Superdex 200 column (GE Healthcare) in 250 mM NaCl, 10 mM tris (pH 8.7), and 2 mM DTT, the AP2 complexes were concentrated to >15 mg/ml with vivaspin concentrators.

Recombinant Cμ2 and other His_6_-tagged proteins were made as in (*47*). Briefly, the proteins were prepared in 500 mM NaCl and 20 mM tris (pH 8.0). Proteins were initially isolated on Ni-NTA agarose, washed with buffer containing 20 mM imidazole, and eluted with buffer supplemented with 300 mM imidazole. GST-tagged Cμ2 and α-appendage proteins were made as in (*51*). Briefly, the proteins were prepared in 200 mM NaCl, 20 mM tris (pH 7.4), and 1 mM DTT. Proteins were initially isolated on GST beads, washed, and cleaved from their tags overnight at room temperature with thrombin. The final purification step for both His_6_-tagged and GST-cleaved proteins involved size exclusion chromatography with a Superdex 200 column (GE Healthcare) in their respective preparation buffers or, in the case of material for ITC, into 150 mM NaCl, 100 mM tris (pH 7.4), and 1 mM DTT. Proteins were subsequently concentrated to >10 mg/ml with vivaspin concentrators. GST-FCHO linkers were similarly purified on GST Sepharose with overnight thrombin cleavage where necessary and lastly by S200 gel filtration with all purification done in HKT buffer [10 mM Hepes, 10 mM tris (pH 7.4), 120 mM potassium acetate, and 2 mM DTT].

### Crystallography

#### Crystallization

Crystals of AP2 FCHO2 chimera were grown in hanging drops with reservoir 18% polyethylene glycol (PEG) 12,000, 0.1 M Na/K phosphate (pH 6.2), 0.2 M NaCl, and 4 mM DTT in the presence of threefold molar excess of InsP6. Crystals were cryoprotected with 20% glycerol.

apo crystals of recombinant histidine-tagged Cμ2 were grown in sitting drops from a mixture of Cμ2 (10 mg/ml) and the FCHO2-derived N1 block peptide (DVDEEGYSIKPETNQNDTKENHFYSS) (2 mg/ml) equilibrating against a reservoir containing 1.5 M ammonium sulfate and 0.1 M Hepes (pH 7.0). Crystals were cryoprotected by soaking in mother liquor supplemented with 20% glycerol and peptide. The N1 peptide was not visible, but Trp^421^ had swung round to partly fill the Y-binding pocket to reduce its solvent exposure (fig. S6, C to E).

Crystals of recombinant histidine-tagged Cμ2 in complex with the FCHO2-derived C block peptide (SDLLAWDPLFG) were grown in sitting drops equilibrating against a reservoir containing 20 mM sodium formate; 20 mM ammonium acetate; 20 mM sodium citrate tribasic dihydrate; 20 mM sodium potassium tartrate tetrahydrate; 20 mM sodium oxamate, imidazole MES monohydrate (pH 6.5), and 20% (v/v) glycerol; and 10% (w/v) PEG 4000 (P3_2_21 crystal form) and in 30 mM magnesium chloride hexahydrate; 30 mM calcium chloride dihydrate, 100 mM sodium Hepes Mops (pH 7.5), and 20% (v/v) ethylene glycol; and 10% (w/v) PEG 8000 (C2 crystal form). The crystals did not require cryoprotection.

Recombinant histidine-tagged Cμ2-FCHO2 chimera crystals were grown in sitting drops against a reservoir containing 20% (w/v) PEG 3350 and 0.2 M dl-malic acid (pH 7.0). The crystals were cryoprotected by soaking in mother liquor supplemented with 30 to 32% glycerol. Recombinant GST-cleaved Cμ2-FCHO2 chimera crystals were grown in sitting drops against a reservoir containing 20% (w/v) PEG 3350 and 0.2 M sodium phosphate dibasic dehydrate (pH 9.1) and were cryoprotected by soaking in mother liquor supplemented with 25% glycerol.

Crystals of AP2 in complex with FCHO2 linker and the DYQRLN peptide derived from TGN38, supplemented with 10 mM KNa tartrate, grew in sitting drops with reservoir of 0.1 M magnesium formate dehydrate and 10 to 15% PEG 3350. The crystals were cryoprotected with 0.1 M Mg formate dehydrate, 13% PEG 3350, 18 to 24% glycerol, and peptide (1 mg/ml). Crystals of AP2 in complex with selenomethionine-labeled FCHO2 linker [made as in (*13*)] and the DYQRLN peptide were grown under similar conditions.

Crystals of α2 ear complexes were grown in sitting drops from a mixture of α2 ear (10 mg/ml) and the FCHO1-derived C block peptide (QSEEQVSKNLFGPPLESAFDHED) (2 mg/ml) against a reservoir containing 1.0 M lithium sulfate and 0.1 M MES (pH 6.5). Crystals were cryoprotected by soaking in mother liquor supplemented with 20% glycerol and peptide.

#### Synchrotron data collection and structure determinations

Diffraction data were collected at Diamond Light Source on beamlines I03, I04, and I24, and data were processed with Xia2. Initial structures were solved by MR with the program PHASER using as search models the previously published structures of Cμ2 (PDB 1BXX), α-appendage (PDB 1W80), and AP2 (PDB 2VGL and 2XA7). The positions of the selenomethionines in the structure of AP2 in complex with TGN and selenomethionine-labeled FCHO2 were determined from the anomalous diffraction collected at a wavelength of 0.92 Å. Iterative rounds of refinement with the programs PHENIX REFINE and REFMAC were interspersed by manual rebuilding of the model with COOT. Crystallographic programs were run from the PHENIX (*78*) and CCP4 packages (*79*), and figures were produced with a commercial version of PyMOL (Schrödinger LLC). Data collection and refinement statistics are in tables S3 to S5.

### Single-particle cryo-EM

AP2-FCHO2 chimera purified as wt AP2 and lastly placed in 50% HKT buffer [10 mM Hepes, 10 mM tris, and 120 mM potassium acetate (pH 7.2)] and 50% core buffer [10 mM tris and 250 mM NaCl (pH 8.7)] was applied to QUANTIFOIL grids of type R1.2/1.3 on 300 copper mesh ± 0.05% β-d-octyl glucoside. Grids were glow-discharged for 60 s at 20 mA using Pelco EasiGlow before application of 3.5 μl of sample (0.4 mg/ml), blotted with Whatman No. 1 filter paper, and plunge-frozen using Vitrobot Mark IV (FEI Company) operated at 4°C and 95% humidity. Data collection was carried out on a Titan Krios transmission electron microscope (FEI/Thermo Fisher Scientific) operated at 300 keV, equipped with a Gatan K3 direct detector (FEI) in counting mode. Automated data acquisition was performed using FEI/Thermo Fisher Scientific EPU software at a nominal magnification of 130,000, which corresponds to a pixel size of 0.362 Å per pixel in superresolution mode. Dose-fractionated movies were acquired using 1.31-s exposures and 48 fractions at a dose of 36.09 *e*^−^/Å^2^ per second in the defocus range of −0.8 to −2.8 μm.

Data collection quality, movie frame alignment, estimation of contrast transfer function (CTF) parameters, particle picking, and extraction were carried out using Warp (*80*). Particle images (718,511) were extracted from 13,250 micrographs with a box size of 340 and imported into CryoSPARC (*81*) for particle curation. 2D classification, ab initio model building, 3D refinement, filtering, and sharpening were also carried out using CryoSPARC as described in the workflow (fig. S5). After iterative 2D classification, 452,076 particles were retained in the dataset, which was subjected to further 3D classification steps. After the first round of 3D refinement, particles assigned to a poorly resolved class and to the Cμ2-out class were removed from the dataset. The remaining particles were further refined to generate the Cμ2-in structure and subjected to further 3D classification to generate the N1-N2–enriched structure. Analysis of particle orientation distribution and assessment of anisotropic resolution by calculating the Fourier Shell Correlation (FSC) within cones of 30° in 3DFSC (*82*) showed that for both structures, the resolution in the *Y* direction was lower than in the other two directions (fig. S5). Random particle subsets in the oversampled views were removed from the datasets using RELION star handler to reduce the resolution anisotropy, and retained particles were further refined to reconstruct the final maps. A total of 143,815 of 347,935 particles were retained for the Cμ2-in structure, and 28,392 of 64,651 particles were retained for the N1-N2–enriched structure. Structures were filtered according to local resolution using CryoSPARC for visualization and interpretation.

The previously determined closed conformation of the AP2 core complex (PDB 2vgl) was fitted into cryo-EM volumes using the phenix.dock_in_map function of the Phenix software suite (CC values reported in table S6). The structures were refined using phenix.real_space_refine (*83*).

### Cryo–electron tomography

#### Liposome preparation

The required mixtures of lipids were assembled in 4:1 chloroform/methanol, dried and rehydrated in HKT buffer, subjected to five freeze-thaw cycles, and extruded 11 times through carbon membranes with 100-nm pores.

#### Cryo–electron tomography sample preparation and data acquisition

AP2 was recruited to liposomes alone or in the presence of the FCHO2 linker by mixing 1.5 μM AP2 or 1.5 μM AP2 and 7.5 μM FCHO2 linker, with 100-nm extruded liposomes (0.2 mg/ml) containing 10% brain PtdIns(4,5)P_2_ and 10% DOPS in a POPC/POPE (3:2) mixture in HKT buffer. Reactions were performed in parallel and incubated for 30 min at 21°C. After incubation, the reactions were supplemented with 1:10 of 10-nm gold fiducial markers in HKT buffer. A total of 3.5 μl of this mixture was applied on a glow-discharged holey carbon grid (CF-2/1-3C, Protochips) and back-side blotted for 4 s at relative humidity of 98% and 18°C, followed by plunge-freezing in liquid ethane (Leica EM GP2 automatic plunger).

Data acquisition was performed as in (*14*). Dose-symmetrical tomographic tilt series (*84*) were collected in a FEI Titan Krios electron microscope operated at 300 kV with a Gatan Quantum energy filter with a slit width of 20 eV and a K3 direct detector operated in counting mode using tilt series controller in Serial EM software. Tilt series contained 41 tilted images (−60° to +60° with 3° increment) with 10-frame movies acquired for each tilt and were imaged with a total exposure of ~130 *e*^−^/A^2^ equally distributed between tilts. The details of data collection are given in table S1.

#### Image preprocessing and tomogram reconstruction

Movie frames were gain-corrected, aligned, and integrated into individual tilt images using align frames (IMOD package) (*85*). Several tilt images and some entire stacks were discarded because of tracking errors during acquisition (table S1). The tilt images were low-pass–filtered according to accumulated dose (*86*), aligned using fiducial markers, and 4× binned tomograms were reconstructed by weighted back projection in Etomo (IMOD) for particle picking purposes. For 3D CTF-corrected tomograms, defocii and astigmatism of individual non–dose-filtered tilts were estimated in CTFPLOTTER (IMOD) (*87*), and phase-flipping CTF correction and tomographic reconstruction were done in novaCTF (*88*) using 15-nm strip width. Tomograms were binned by two, four, and eight times (hereafter called bin2, bin4, and bin8 tomograms) with anti-aliasing.

#### Subtomogram alignment

Subtomogram alignment, averaging, and classification were done as previously described in (*14*) in subTOM packages (www2.mrc-lmb.cam.ac.uk/groups/briggs/resources and https://github.com/DustinMorado/subTOM/releases/tag/v1.1.4). Dynamo (*89*) and Relion (*90*) packages were used for mask making.

#### Picking initial subtomograms and alignment to the membrane

Initial position was picked as described previously (*14*) using the “pick particle” Chimera plug-in (*91*), approximating liposomes by spheres with uniform surface sampling at every 42 Å. The particles were oriented normally to the sphere surface with random in-plane angle. Subtomograms were extracted at these geometrically defined positions and orientations from bin8 tomograms and averaged producing initial models. The membrane alignment of subtomogram was then refined by allowing only shifts normal to the membrane and a conical angular search range of ±30°. Particles that failed to align to the membrane were removed on the basis of a cross-correlation threshold selected manually for each tomogram.

#### Subtomogram averaging of AP2

The previously published EM map of AP2 on tyrosine cargo-containing membranes (*14*) was low-pass–filtered to 42 Å and used as a reference to find initial AP2 positions. Starting from the initial positions and orientations determined by membrane alignment, bin4 subtomograms were aligned to this reference with lateral shift limited to 80 Å to confine the alignment within the area occupied by a single AP2. Where subtomograms had converged to positions within 70 Å of one another, we selected the subtomogram with the highest cross-correlation score and discarded all others. Subtomograms were further subjected to classification in the subTOM package to remove “empty” subtomograms. The remaining subtomograms were then sorted into identically sized odd and even subsets by vesicle, and iterative subtomogram alignment and averaging were performed in bin2 and then in bin1, gradually decreasing the search space and increments for angular and spatial parameters, and moving the low-pass filter toward higher resolution. Upon alignment convergence in bin2, particles that had diverged from membrane alignment were removed using a cross-correlation threshold defined manually for each tomogram. Table S2 shows data processing statistics.

### Brain cytosol and HeLa cell extract preparation

Rat brain cytosol was prepared in a homogenization buffer of 25 mM Hepes-KOH (pH 7.2), 250 mM sucrose, 2 mM EDTA, and 2 mM EGTA. Rapidly thawed frozen brain tissue was homogenized in a Waring blender at 4°C in the presence of homogenization buffer with 2 mM phenylmethylsulfonyl fluoride, 5 mM benzamidine, and complete protease inhibitor tablets. The resulting thick homogenate was centrifuged at 15,000*g* for 20 min, and the postnuclear supernatant fraction was recentrifuged at 17,500*g* for 20 min. The supernatant was then centrifuged at 105,000*g* for 60 min. The resulting high-speed supernatant (cytosol) was stored in small frozen aliquots at −80°C.

HeLa cell Triton X-100 lysates were prepared by first collecting confluent cells from petri dishes using Cellstripper and centrifuging at 500*g* for 5 min. The cell pellet was resuspended in cold 25 mM Hepes-KOH (pH 7.2), 125 mM potassium acetate, 5 mM magnesium acetate, 2 mM EDTA, 2 mM EGTA, 2 mM DTT, and 1% Triton X-100 and incubated on ice for 30 min with occasional mixing. After centrifugation at 24,000*g* for 20 min to remove insoluble material, the supernatant (lysate) was stored in frozen aliquots at −80°C. Thawed cytosol/lysate samples for assays were centrifuged at 125,000*g* for 20 min at 4°C immediately before use.

### GST pull-down assays

A measured amount of GST or GST-fusion protein was immobilized onto 60 μl of 50% slurry of GSH Sepharose beads in microfuge tubes and the volume made up to 750 μl with buffer. After incubation at 4°C with continuous mixing, the Sepharose beads were recovered by centrifugation (10,000*g* for 1 min), and the supernatants were removed. Each bead pellet was washed three times with cold assay buffer, and most of the buffer was carefully aspirated, leaving a final equivalent volume of ~50 μl.

Using cytosols, aliquots of rat brain cytosol or HeLa cell Triton X-100 lysate were thawed and centrifuged at 125,000*g* for 20 min at 4°C to remove insoluble particulate matter before addition of 200 μl to the tubes of immobilized GST or GST-fusion protein and then incubated at 4°C for 60 min with continual mixing. Binding assays were terminated by centrifugation (10,000*g* for 1 min at 4°C), and 60 μl was removed and transferred to a new microfuge tube. The pellets were then washed three times by centrifugation with 1 ml per wash of ice-cold buffer, and the supernatants were aspirated and discarded. The Sepharose beads were resuspended in reducing SDS sample buffer, and the volumes were adjusted manually so that all tubes were equivalent. After boiling and centrifugation (12,000*g* for 1 min), aliquots of samples were fractionated by SDS-PAGE and stained either with Coomassie brilliant blue or blotted with indicated antibodies.

### Isothermal titration microcalorimetry

Experiments were performed using a Nano ITC machine from TA Instruments. For ITC, proteins were gel filtered into 100 mM tris (pH 7.4), 150 mM NaCl, and 0.25 mM TCEP. Peptides were dissolved in the same buffer. PHear domains at concentrations between 0.10 and 0.15 M were placed in the cell at 12°C, and peptides at concentrations between 1 and 5 mM (depending on a peptide) were titrated with 20 injections of 2.43 μl each separated by 2.5 min. A relevant peptide-into-buffer blank was subtracted from all data, and for constructs, which displayed measurable binding, a minimum of three independent runs that showed clear saturation of binding were used to calculate the mean *K*_D_ of the reaction, its stoichiometry (*n*), and their corresponding SEM values. Analysis of results and final figures was carried out using the NanoAnalyzeTM software.

### BLI experiments

Real-time kinetic measurements of AP2 core binding to immobilized GST-FCHO1 linker and GST-FCHO2 linker at 25°C were conducted using an Octet RED96 (Pall FortéBio). AP2 concentrations varied by twofold dilution series (13.5 to 0.8 and 10.0 to 0.2 μM, respectively). Samples and buffers were dispensed into 96-well microtiter plates (Greiner) at a volume of 200 μl per well. Anti-GST biosensors (Pall FortéBio) were incubated in the specific assay buffer for at least 30 min before loading with the appropriate GST-FCHO linker for 5 min, followed by blocking with GST-ARRS control for 10 min. Negative controls were analyzed with biosensors uniquely loaded with the GST-ARRS control. Every binding experiment consisted of three steps: incubation for 10 min in the specific assay buffer, followed by 15 min of incubation with AP2 in the same buffer (association phase), and then by 10 min of incubation with the same buffer without AP2 to measure AP2 off rate (dissociation phase). Nonspecific binding was reduced by supplementing buffers with 0.1% NP-40 and bovine serum albumin (1 mg/ml).

A similar experimental setup was used in the analysis of AP2 core and (AP2:μ2FCHO2-N1+N2+N3) chimera binding to immobilized GST-TGN. The negative control was GST-Control. Binding was measured for the same concentrations in parallel against both the TGN38-loaded and the control-only–loaded biosensors. Twofold dilution series of AP2 core and AP2 FCHO2 chimera were analyzed (3.3 to 0.1 and 2.6 to 0.9 μM, respectively). Because of slower on and off rates, association times were increased to 20 min and dissociation times to 25 min.

Data were processed with the Octet Data Acquisition 7.0 software, individually fitted using ForteBio Analysis software 7.0, and subsequently further analyzed with the PRISM software (GraphPad) to determine equilibrium dissociation constants (*K*_D_). The maximum binding capacity of each biosensor was scaled with respect to each other, according to the molecular weight of the AP2 analyzed.

### Liposome spin-downs

GST-FCHO2 WT linker or a nonbinding control (GST-ARRS control) at a final concentration of 30 μM was incubated at 4°C for 30 min with AP2 core at a concentration of 2.5 μM, in 95% HKT buffer with lysozyme (0.2 mg/ml) as a “carrier,” AEBSF (25 μg/ml), and 5 mM DTT. Core buffer (contributed by the purified AP2) was restricted to 5% of the volume. The mixture was then centrifuged briefly to remove any insoluble material. Samples were removed and added to either an equal volume of liposomes [containing phosphatidylcholine (PC)/phosphatidylethanolamine (PE), PC/PE/PS/PtdIns(4,5)P_2_, or PC/PE/PS/ PtdIns(4,5)P_2_/TGN38] or an equal bed volume of GSH Sepharose beads prewashed into HKT buffer. Final concentrations were therefore 15 μM (GST-FCHO2/ARRS) and 1.25 μM AP2. The mixtures were incubated for 30 min at 21°C with continuous gentle inversion, then the liposomes were pelleted by centrifugation, supernatants were removed, and the pellets resuspended in an equal volume before the addition of SDS loading dye and analysis by SDS-PAGE. The GSH Sepharose beads were washed three times with 1 ml of HKT supplemented with lysozyme (0.2 mg/ml), AEBSF (25 μg/ml), and 5 mM DTT, then resuspended in the same buffer, and analyzed by SDS-PAGE. All experiments were done in triplicate.

## Notes

View/request a protocol for this paper from *Bio-protocol*.
