# Supplementary material for: FCHO controls AP2’s initiating role in endocytosis through a PtdIns(4,5)P2-dependent switch
Source: Sci Adv. 2022 Apr 29;8(17):eabn2018. doi: 10.1126/sciadv.abn2018 (PMC9054013; doi:10.1126/sciadv.abn2018)
Supplement: Supplementary file 1 — Figs. S1 to S10 Tables S1 to S6 [file sciadv.abn2018_sm.pdf]

Science Advances
AAAS

Supplementary Materials for

FCHO controls AP2's initiating role in endocytosis through a PtdIns(4,5) P_2-dependent switch

Nathan R. Zaccai, Zuzana Kadlecova*, Veronica Kane Dickson, Kseniya Korobchevskaya, Jan Kamenicky, Oleksiy Kovtun, Perunthottathu K. Umasankar, Antoni G. Wrobel, Jonathan G. G. Kaufman, Sally R. Gray, Kun Qu, Philip R. Evans, Marco Fritzsche, Filip Sroubek, Stefan Höning, John A. G. Briggs, Bernard T. Kelly, David J. Owen*, Linton M. Traub

*Corresponding author. Email: zk241@cam.ac.uk (Z.K.); djo30@cam.ac.uk (D.J.O.)

Published 29 April 2022, *Sci. Adv.* **8**, eabn2018 (2022)
DOI: 10.1126/sciadv.abn2018

The PDF file includes:

Figs. S1 to S10
Tables S1 to S6
Legends for movies S1 to S4

Other Supplementary Material for this manuscript includes the following:

Movies S1 to S4

Zaccai, Kadlecova Figure S1

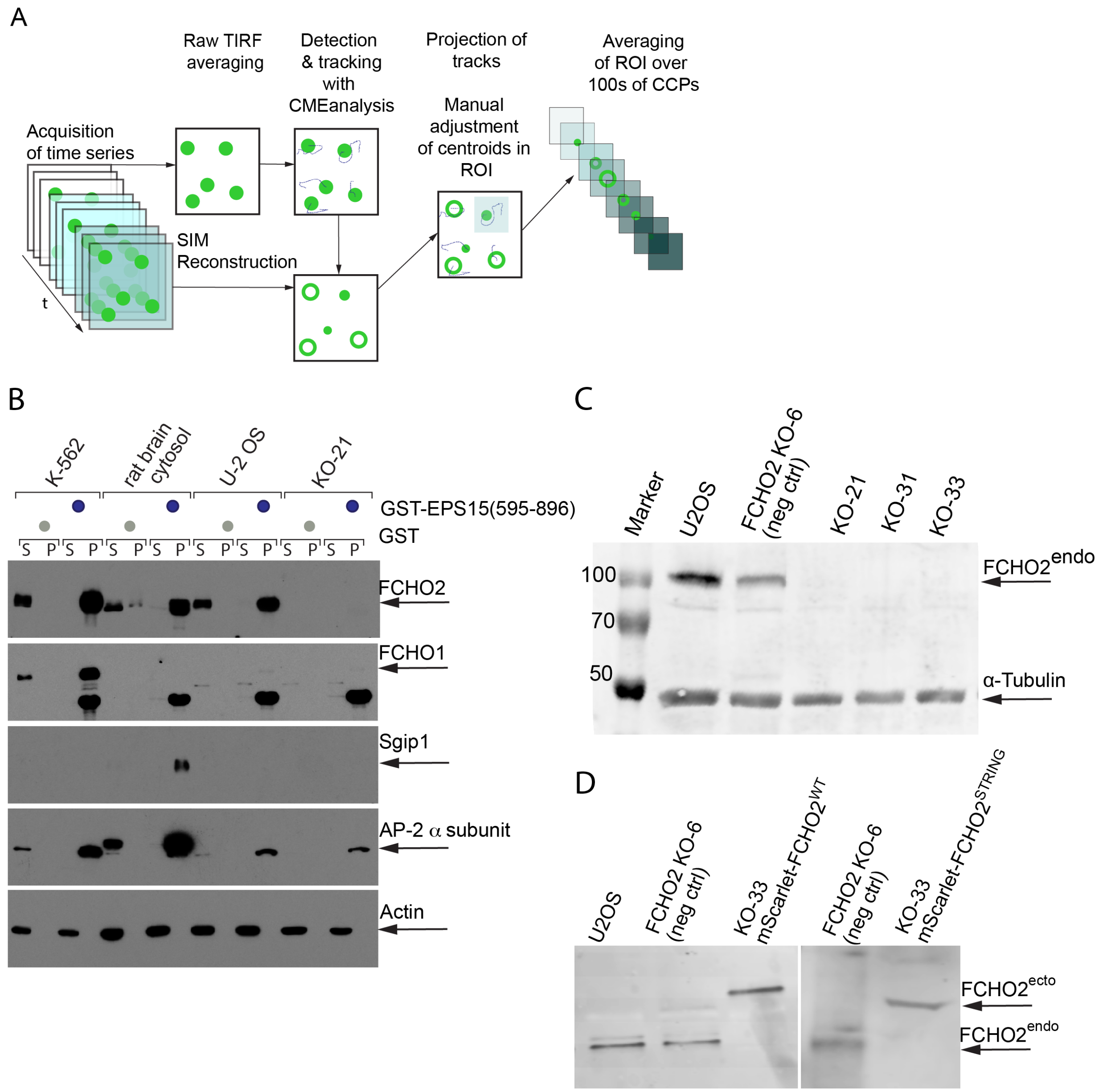

Fig.S1 Validation of FCHO2 levels in U-2 OS cells.

A Simplified scheme of the analytical workflow used for characterizing CCP formation in live cell eTIRF-SIM movies: U-2 OS cells expressing fluorescently tagged mScarlet-FCHO2^{WT} and egfp-CLCa at near endogenous levels were imaged with eTIRF-SIM in which nine raw TIRF images in the 9 orientations of the grid pattern are acquired to produce a reconstructed super-resolved TIRF-SIM image with about 110nm resolution in both channels. The detection and tracking were obtained for individual CCPs with CMEAnalysis package from the averaged raw TIRF images prior to reconstruction. The centroids of CCPs and tracks were manually aligned simultaneously in both channels to allow ROI extraction and downstream analysis (see M&M). These ROI were averaged for individual CCPs and lifetime phases of CCP formation.

B Western blot analysis of the protein quantity of the three FCHO paralogues (muniscins): FCHO1, FCHO2 and Sgip in U-2 OS, U-2 OS FCHO2 KO clone 21, K-562 and rat brain cytosol.

C Validation of isogenic U-2 OS FCHO2 knockout clones 21, 31 and 33 and negative control clone 6 by western blot.

D Western blot validation of FCHO2 reconstitution in FCHO2 knockout clone 33 by retrovirus mediated expression of mScarlet-FCHO2^{WT} or mScarlet-FCHO2^{STRING} at near-endogeneous levels.

Zaccai, Kadlecova Fig.S2

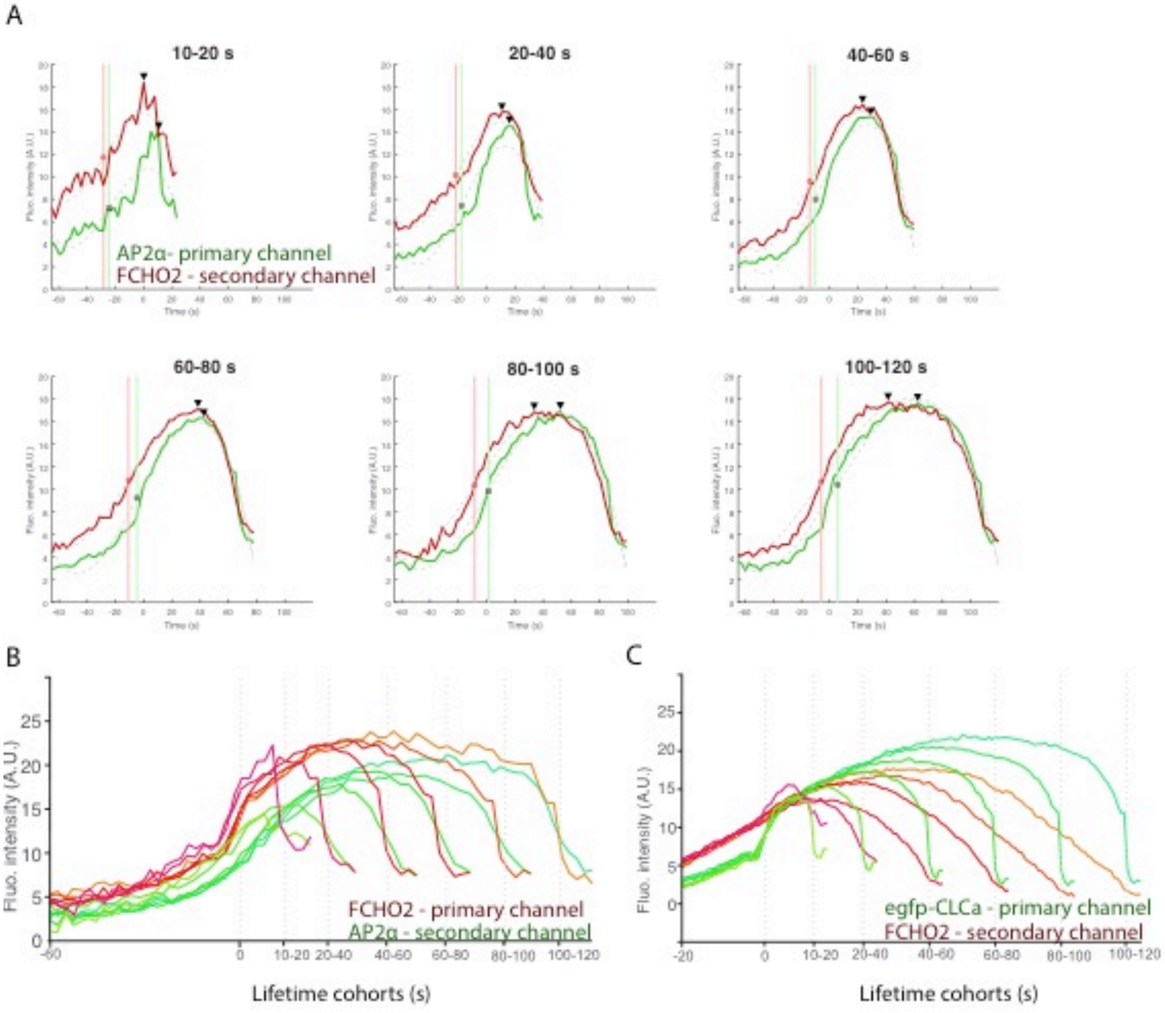

Fig.S2 Live cell TIRF microscopy in engineered U-2 OS cells.

A Average AP2 (green) and FCHO2 (red) fluorescence intensity traces in lifetime cohorts of FCHO2-positive CCPs. Average fluorescence intensity was plotted for 25 frames prior to the first detected time point in the reference AP2 channel. This allowed visualizing initial FCHO2 intensities in the same pixel before AP2 or egfp-CLCa appearance. Black triangles indicate maximum intensity time point. Red circle indicates inflection timepoints based on cubic polynomial approximation of cohorts (grey dashed lines) for FCHO2 trace. Green circle indicates inflection time point for AP2.

B Average FCHO2 (red tones) and AP2 (green tones) fluorescence intensity traces in CCP lifetime cohorts. In this analysis FCHO2 was chosen as the reference channel to define the intensity time courses of AP2 at the time points and locations defined by FCHO2 signal detection. 96% of all dynamic FCHO2 assemblies were classified as positive for AP2.

C Average FCHO2 (red tones) and egfp-CLCa (green tones) fluorescence intensity traces in CCP lifetime cohorts.

Zaccai, Kadlecova Figure S3

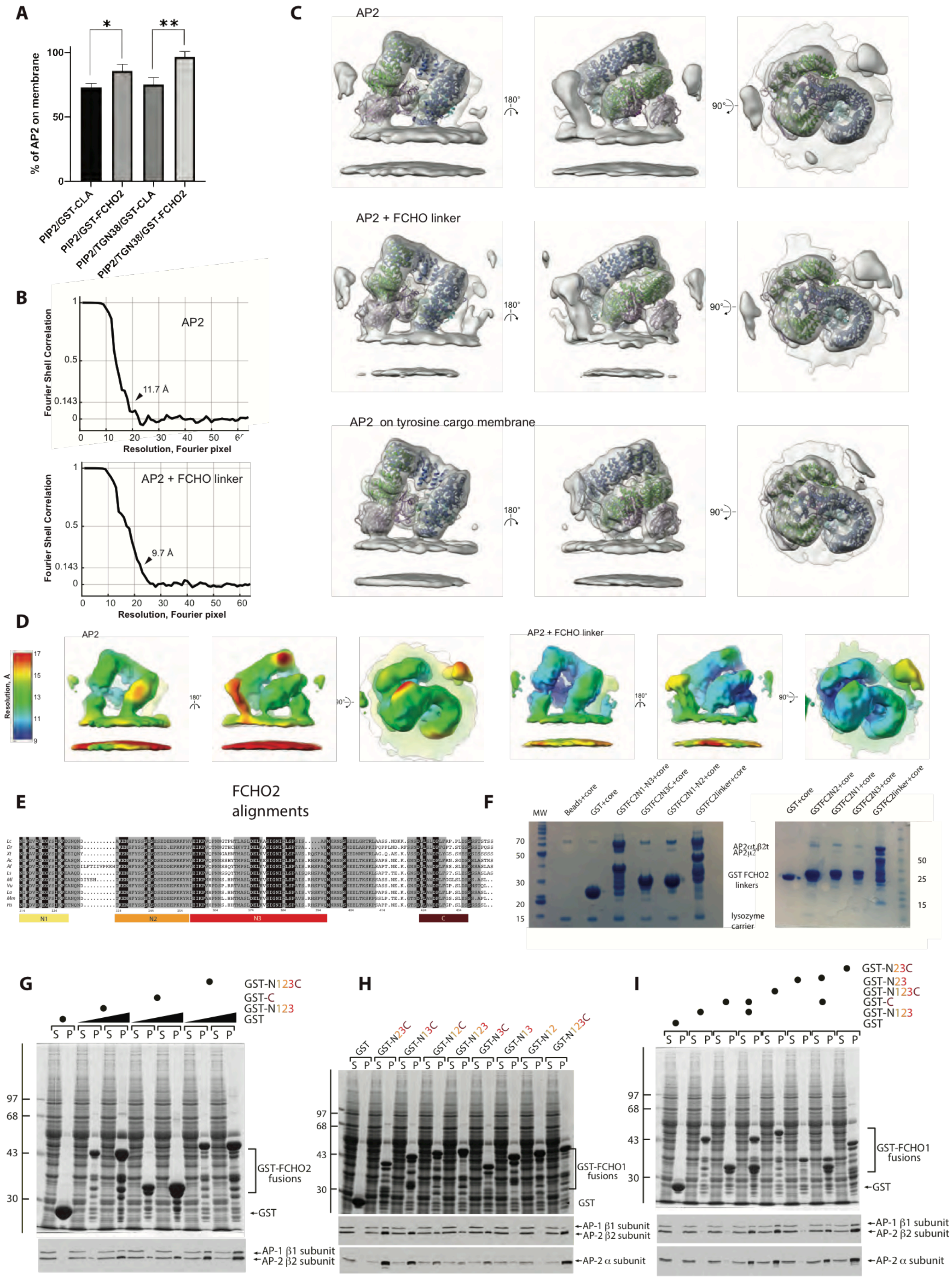

Fig.S3 AP2 contains overlapping binding sites for FCHO linker and PtdIns4,5P$_2$

A Comparison of AP-2 recruitment to liposomes in the presence and absence of GST-FCHO2. Liposomes (either PC/PE/PtdIns(4,5)P$_2$/PtdSer or PC/PE/PtdIns(4,5)P$_2$/PtdSer/YXXØ cargo peptide) were incubated with mixtures of GST-FCHO2 or GST-ARRS (a non-binding control) and AP-2, the liposomes were pelleted by centrifugation and protein present in the pelleted and supernatant fractions analyzed by SDS-PAGE. The percentages of AP-2 recruited to each type of liposome (assessed by densitometry) are shown as means ± standard error on the mean (3 independent experiments). By Student's t-test, the percentage of AP-2 recruited was increased by the presence of GST-FCHO2 for both liposome types (PtdIns(4,5)P$_2$, $p = 0.023$; PtdIns(4,5)P$_2$/ YXXØ cargo, $p = 0.006$).

B Comparison the conformations of membrane-recruited AP2. Cryo-EM maps for AP2 on membrane without cargo in the absence or presence of five-fold molar excess of the FCHO2 linker, and the previously published structure of AP2 bound to YxxΦ motif containing membranes in the absence of FCHO2 (EMDB-10748). The cryo-EM maps are filtered to 13 Å and all are fitted with the ribbon model of AP2 on the YxxΦ motif containing membranes (PDB: 6YAF). In all cases AP2 is recruited in its open state and there are no observable differences in the conformations at the given resolution.

C,D Global and local resolution of on-membrane AP2 EM maps. **C**, EM maps colored by local resolution for both structures determined in this study and **D**, corresponding FSC plots with arrows indicating the measured global resolution at the 0.143 threshold.

E Alignment of FCHO2 linker region from species spanning ~400 million years with most distantly related species to humans shown at top. Coelacanth Lc; Zebrafish Dr fcho2; Xenopus

Xt fcho2; Anole Chameleon Ac fcho2; Emperor penguin Af; Society finch Ls; Brown bat Ml; Wombat Vu; Elephant La; Mouse Mm; Human Hs. Identities are shown in black and similarities in grey. The coloured boxes denote the N1, N2, N3 and C block definitions as defined in the human FCHO2 protein and are used throughout the work.

F Binding of constructs of GSTFCHO2 linkers containing conserved sequence blocks (as in Fig. 4A and S3E) to recombinant AP2 cores. 30µg of GST or GST-FCHO2 linker fusion proteins as indicated were immobilised onto 60µl of 50% slurry of glutathione-Sepharose beads. Binding was carried 750ul for 30minutes at 4°C with continuous mixing. Sepharose beads were washed 3x 1ml buffer and run on a 12.5% SDS PAGE gel with MW markers. The gel was stained with Coomassie blue: data is summarised in Fig. 4B. Removal of C block has little effect on AP2 core binding. Removal of any further block causes a dramatic reduction in binding. Single blocks bind only very weakly.

G Binding of 10µg (left) and 30µg (right) of the indicated GSTFCHO2 linkers (Fig. 4A, S3E) to AP2 contained in brain cytosol as in **F** and detailed in Methods: Upper panel Coomassie staining of SDS PAGE gels and lower panel blotting with anti $\beta1/\beta2$ antibody. S soluble fraction. P pellet fraction. Data is summarised in Fig. 4B deletion of C block reduces binding to AP2 when compared with full length linker and C block retains some AP2 binding.

H, I Binding of GSTFCHO1 linker constructs containing conserved sequence blocks as indicated in Fig. 4A, S3E to AP2 derived from cell cytosol. (**H**) uses single constructs and (**I**) uses combinations of the same as indicated. Binding experiments were carried out as in **G** and outlined in Methods Upper panels Coomassie staining of SDS PAGE gels; middle and lower

panels blotted with anti $\beta 1/\beta 2$ antibody and anti α antibody. S soluble fraction. P pellet fraction.

H Deletion of any one block has minor effects on the binding of the FCHO1 linker whereas deletion of more than one block severely compromises binding**:** All blocks show some very weak binding to intact brain cytosol derived AP2. **I** Combinations of various sequence blocks (N1+N2+N3) and C or (N2+N3) and C are more than additive for binding to the individual block constructs indicating cooperativity i.e. avidity effects are occurring

Zaccai, Kadlecova Figure S4

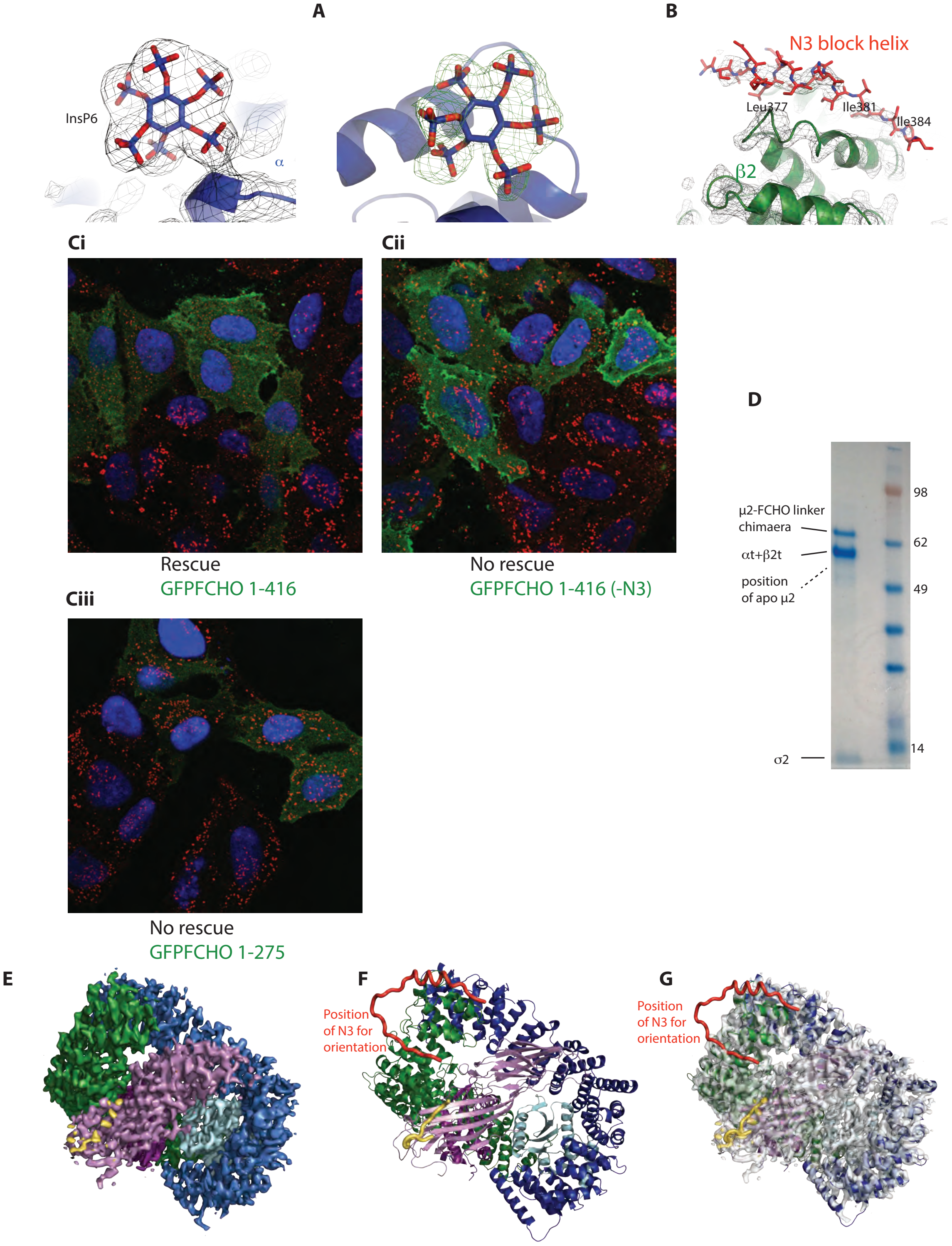

Fig.S4 FCHO2 linker can bind intramolecularly to closed AP2.

A Left hand panel 2.9Å resolution 2Fo-Fc electron density of D-myo-inositol-1,2,3,4,5,6-hexakisphosphate (InsP6; PtdIns4,5P2 analogue) bound to the a subunit (blue) of AP2/FCHO chimaera in closed conformation contoured at 1σ. During molecular replacement, InsP6 was not included in the search model. Right hand panel – Fo-Fc omit map for InsP6 contoured at 3σ (green electron density) calculated after simulated annealing with the InsP6 occupancy set to zero.

B 2.9Å resolution 2Fo-Fc electron density contoured at 1σ of the AP2/FCHO chimaera in closed conformation, showing the N3 block helix (red) bound to β2 subunit (green).

C HeLa 1E cell lines, deleted for FCHO1 and FCHO2 (*15*) transfected with constructs encoding GFP N-terminally appended to portions of FCHO2 as indicated. Transfection with GFP FCHOFBAR+wt linker (residues 1-416) rescues the fewer, large irregular AP2-containing CCPs patch phenotype caused by FCHO deletion and described in (*15*) (i) to an increased number of standard sized CCPs. Constructs comprising the BAR+ linker (residues 1-416) but with the linker N3 block deleted and replaced by an unstructured polypeptide (GFPFCHOBAR+linker ΔN3) (ii) and the BAR domain of FCHO only (residues 1-275) (iii) fail to rescue the CCP phenotype as in (*15*). GFP constructs are in green, AP2-containing clathirn-coated structures shown in red and the nucleus indicated in blue. These data indicate that the presence of N3 block of the linker seen by crystallography (Figs.3A and S4B) bound to β2 trunk is important for FCHO2 function in respect to AP2 binding and CCP morphology *in vivo* despite N3 being only one of four FCHO linker:AP2 contact sites and that on its own N3 displays only minimal binding so reaffirming the

importance of avidity in the interaction: These data demonstrate transfer of data from structure back to cell biology

D Coomassie stained SDS PAGE gel of AP2:μ2FCHO2linker following placing on grids. The upper band (~70kDa) of the chimaeric μ2 band shows no degradation and there is no 50kDa band corresponding to apo μ2 (i.e. with no appended FCHO linker): Together these imply there has been no degradation or cleavage of the construct in solution suggesting that the apparent absence of Cμ2 in ~17% of particles is due to its static disorder following its displacement from AP2 bowls once they have been transitioned into an open conformation due to FCHO linker induced destabilization

E Cryo-EM reconstruction (3.8 Å) of AP2:μ2FCHO2-N1+N2+N3 chimera majority class. The map is a result of a reconstruction of the highest uniform population of particles (Cμ2-in) (see also Fig. S5). The AP2 is in the closed conformation and is coloured according to the underlying subunits with the position of the FCHO linker N1 block indicated in yellow.

F Ribbon representation in same orientation and colour scheme as **E**. For orientation purposes only, N3, which is not seen in SPA is shown in red, in thin worm representation in the position defined by X-ray crystallography (Fig. 5).

G Structure as in **F** overlayed with semi-transparent, white Cryo-EM reconstruction (3.8 Å).

Zaccai, Kadlecova Figure S5

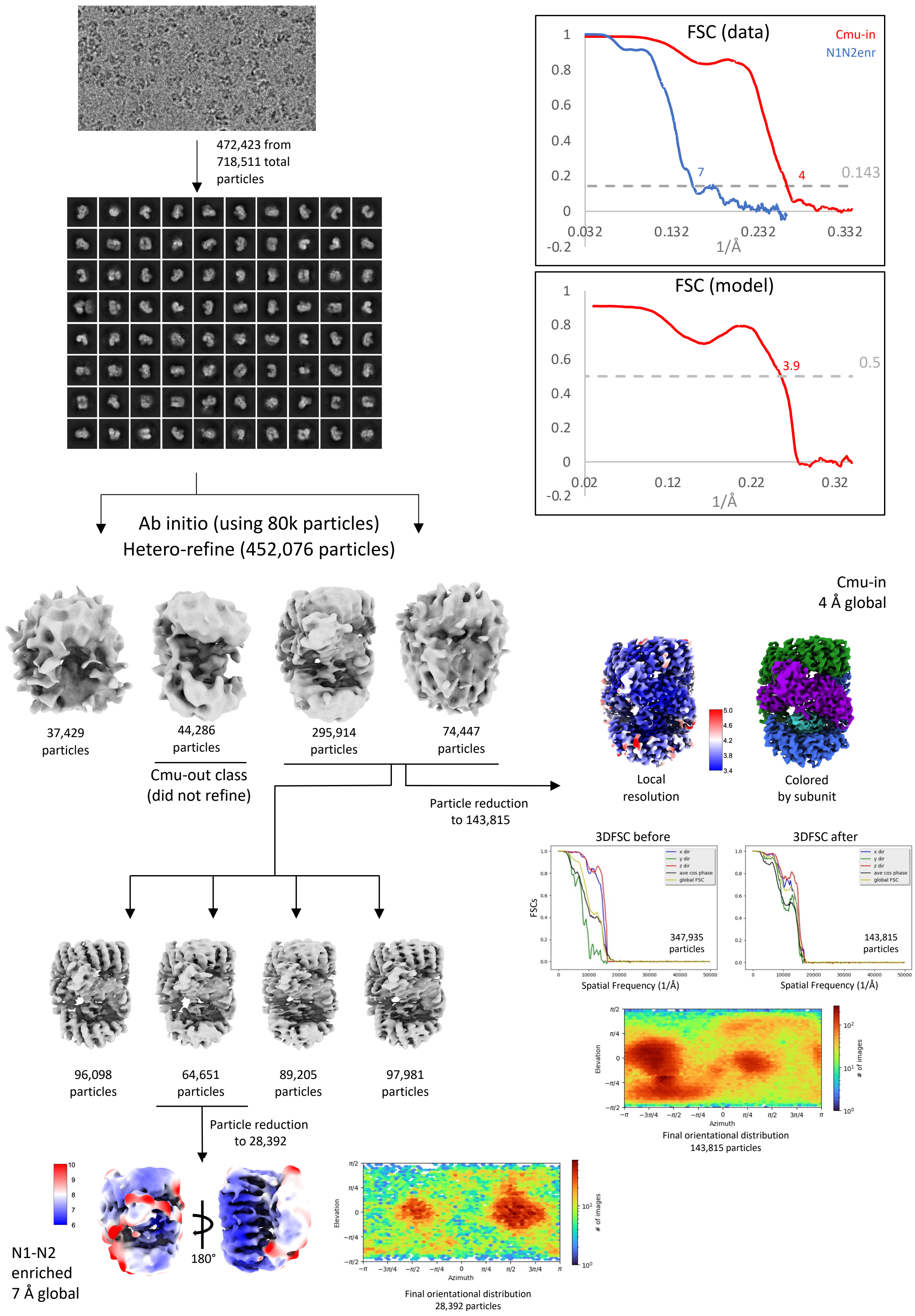

Supplemental Figure S5. Single particle cryo-EM image processing workflow.

AP2 particles were automatically picked and extracted from micrographs following motion and contrast transfer function correction. Particles were first subjected to 2D classification and classes with poor resolution or appearance were removed before *ab initio* models were determined using cryoSPARC and all particles were sorted into 4 classes. The majority class, a pool of two subclasses resembling closed AP2 core, was refined and also further sub-classified into 4 populations, each with small amounts of density outside of that expected for AP2 core alone both in the region of FCHO2 N1 and in the region adjacent to Cμ2. One class had density continuous with FCHO2 N1 (location determined by crystallography), shown here as the N1N2-enriched subclass. Final structures for the Cμ2-in structure and the N1N2-enriched subclass were generated after particle reduction (see also Methods section) as mitigation for preferential orientation as determined using 3DFSC (see directional FSC plots for the Cμ2-in structure before and after particle reduction, inset) followed by non-uniform refinement. The final orientational distribution plots generated by cryoSPARC are shown for both structures. Reconstructions of the overall Cμ2-in structure and the N1N2-enriched structure are illustrated with estimated local resolution. Local filtering was applied to the refined Cμ2-out map after local resolution estimation. FSC curves for half-maps of each refined structure are shown in the top right panel, as well as that of the model vs data for the overall Cμ2-in structure.

Zaccai, Kadlecova Fig.S6

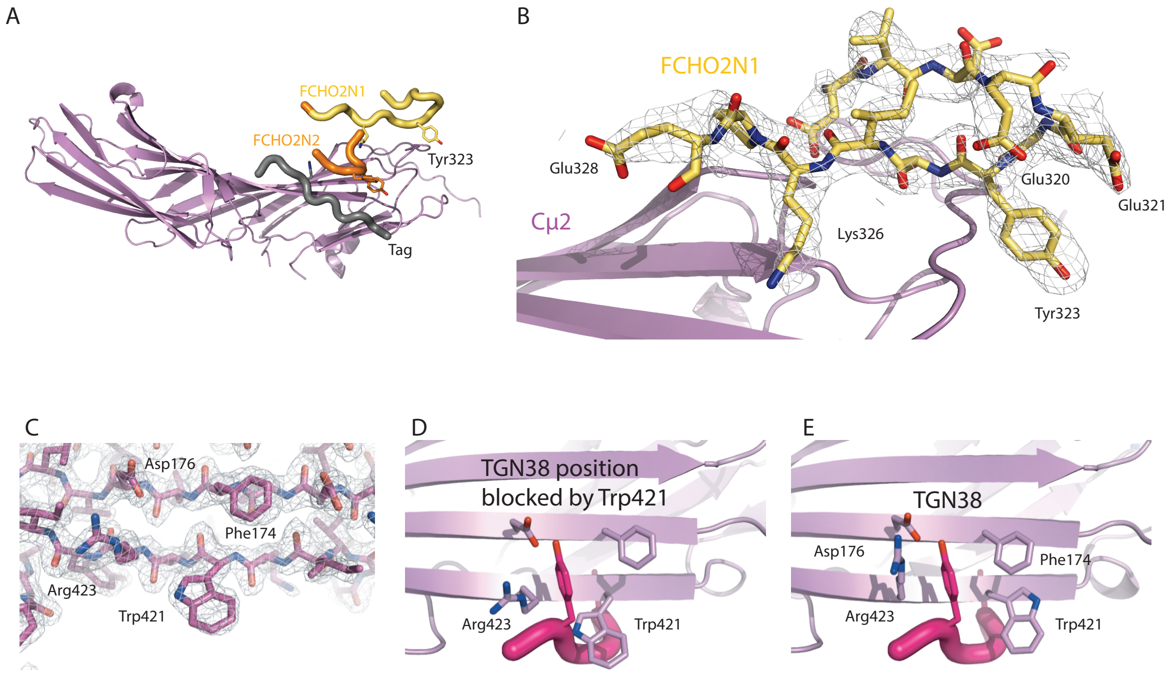

Fig.S6 Structures of Cμ2:FCHO2-N1+N2 chimaeras

A 2.3Å resolution overall structure of His_6-tagged Cμ2:FCHO2-N1+N2 chimaera: N1 (yellow) stays in the same position as in GST cleaved tag structure shown in Fig.6E: the main difference to cleaved GST tagged version is the position of the part of N2 (orange).

B 2.6 Å-resolution 2Fo-Fc electron density of Cμ2:FCHO2-N1+N2 chimaera (cleaved GST tag) contoured at 1.3σ showing molecular details of the binding of N1.

C, D, E, In the absence of YxxΦ ligand or a bound back affinity tag assuming its position, μ2Trp421 'swings back' into the hydrophobic Y pocket to shield it from solvent. **C** 1.9Å resolution 2Fo-Fc electron density contoured at 1.5σ showing μ2Trp421 'swung back' **D** compares this new position for μ2Trp421 with that of a standard YXXØ motif from published structures of complexes between Cμ2 and YXXØ motif peptides **E** : the μ2-Trp421 side chain is rotated through angles χ1 by ~110° and χ2 by ~130°, i.e. it is flipped back as compared to its position in a YXXØ motif liganded structures.

Zaccai, Kadlecova Fig.S7

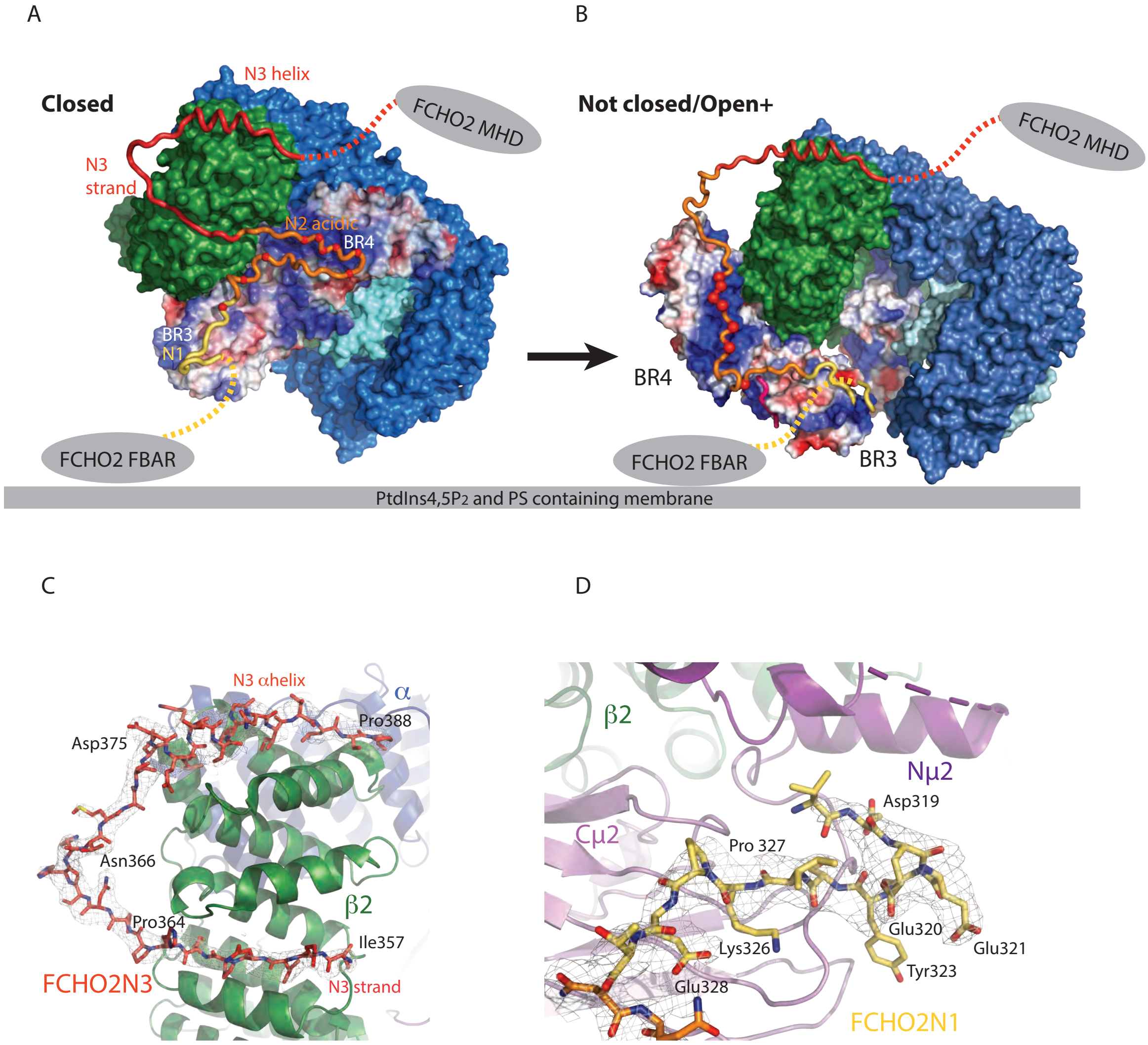

Fig.S7 FCHO linker binds to β2 and µ2 subunits of AP2 using blocks N3 and N1 respectively

A Model of the closed of AP2 core bound to FCHO2-N1N2N3. The FCHO2 blocks and the AP2 α, β2 and σ subunits are coloured as previously (N1 yellow, N2 orange, N3 red, α blue, β2 green and σ2 cyan). The Cμ2 is shown in electrostatic surface representation to demonstrate how the FCHO2 acidic region (acidic residues as red spheres) could interact with the positively charged surface of Cμ2. The closed AP2 is loosely bound to the PM only via it's a PtdIns4,5P_2-binding site.

B Once bound to closed AP2, the N1-N3 linker could destabilise the closed conformation by interfering with the Cµ2 / β2 subunit interface in the region where YxxΦ cargo binds to β2Val365 and β2Tyr405. Once the AP2 'bowl' changes shape the Cµ2 will be ejected and could take up a number of positions, which we believe are averaged out in our SPA (Fig. 5B, S5) in which the YxxΦ cargo binding site is free (in line with data in Fig. 7) – one such possible conformation of AP2 is that which we have previously designated as Open+, which is modelled bound to FCHO2 linker here such that N, N2 and N3 can all bond simultaneously but Cµ2 is not membrane bound but AP2 is loosely bound to the PM via it's a PtdIns4,5P_2-binding site.

C, D 3.3Å resolution 2Fo-Fc electron density for the FCHO2 linker bound to open AP2 contoured at 1σ. (**C**) N3 block (red) bound to β2 subunit (green) (**D**) N1 (yellow) bound to Cµ2 (purple): important side chains in the FCHO linker side of the binding are indicated.

Zaccai, Kadlecova Figure S8

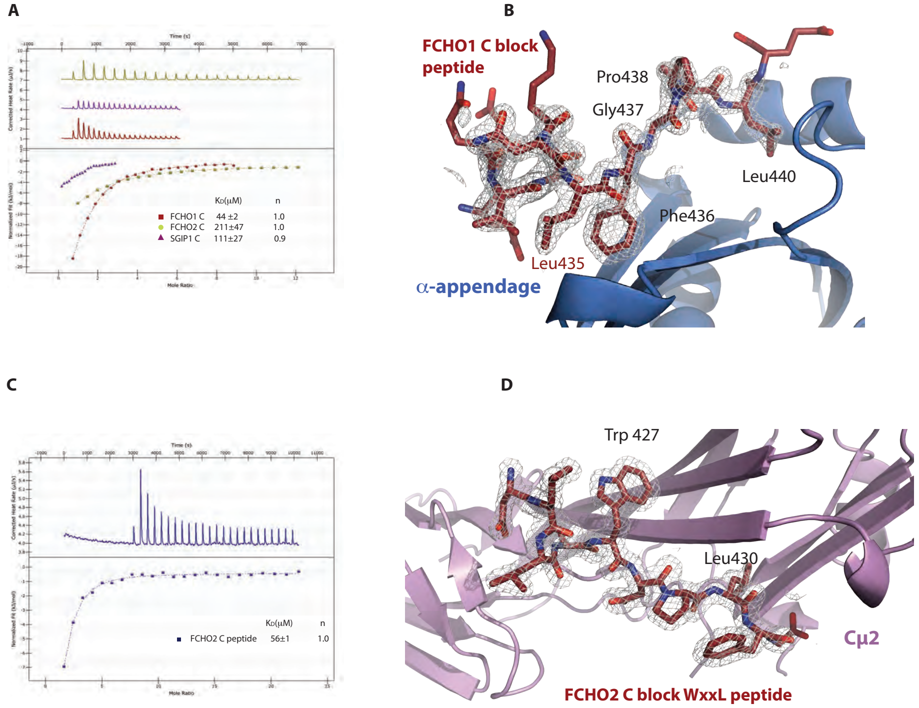

Fig.S8 Details of FCHO C block binding to α-appendage and to Cµ2

A, B FCHO1 C block (claret) bound to AP2 α-appendage (blue). **A** Isothermal Calorimetry analysis and resultant K_Ds of C block peptides of FCHO1 (red K_D ~40µM), FCHO2 (green K_D ~200µM) and SGIP (purple K_D ~110µM) binding to α-appendage. **B** 1.4 Å resolution 2Fo-Fc map contoured at 1.6σ. An apo structure of the α-appendage was used as MR search model (PDB 1W80 without ligands) – the direction of the peptide chain is clearly opposite to that of the published DP[FW], FxDxF motif/α-appendage complexes.

C, D FCHO2 C block (claret) in the YxxΦ binding site on Cµ2 (purple). **C** Isothermal Calorimetry analysis of FCHO2 C block peptide binding to AP2 Cµ2 Resultant K_D ~50µM (blue): the tightest binding cargo YxxΦ peptide is TGN38 (K_D~2µM) with standard cargo binding having K_Ds between 30 and 100µM (*54*). No binding was observed for FCHO1 and SGIP C block peptides. **D** 1.7Å resolution 2Fo-Fc map contoured at 1.6σ. An apo structure of Cµ2 was used as MR search model (PDB 1BXX without ligands).

Zaccai, Kadlecova Fig.S9

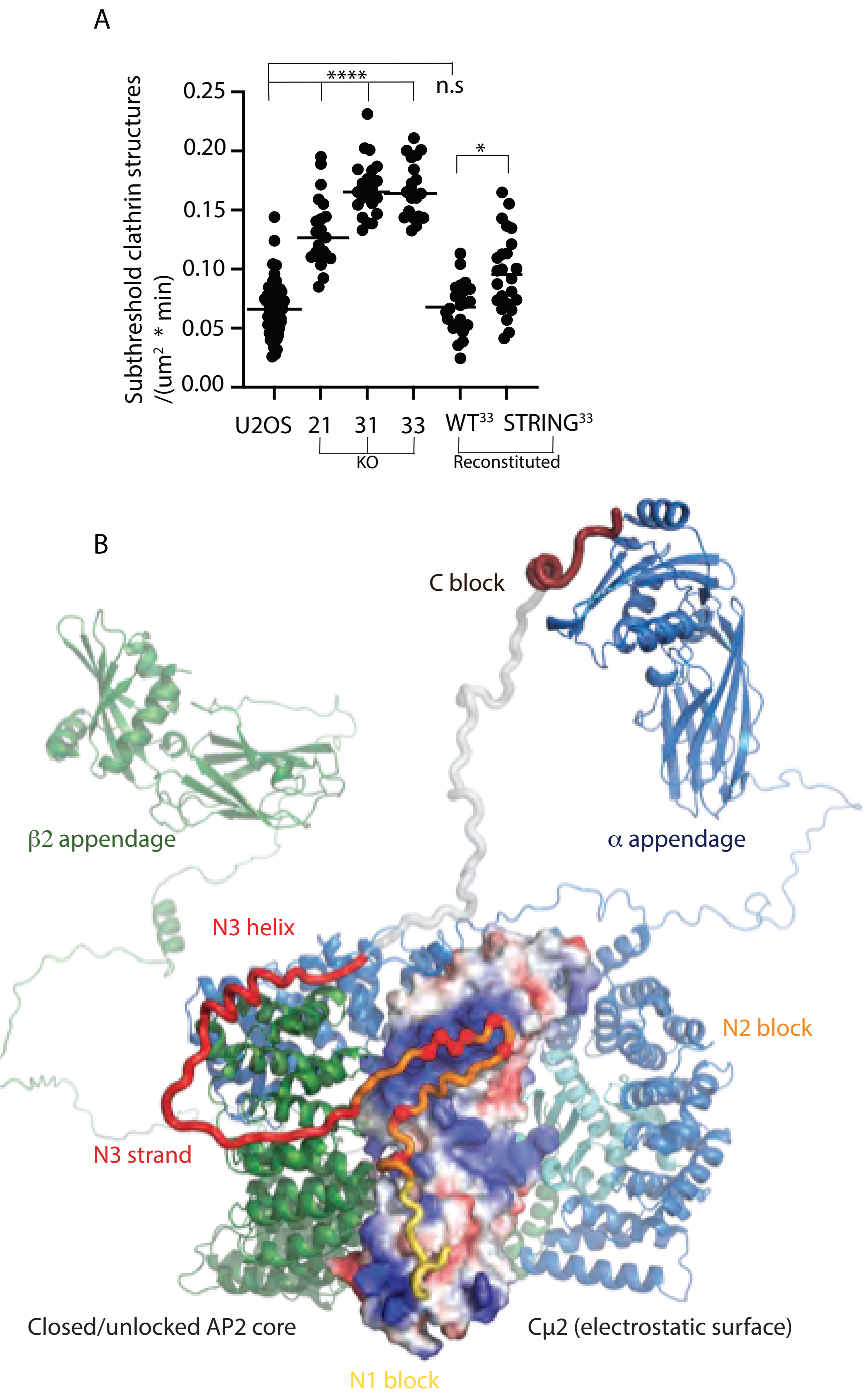

Fig.S9 FCHO linker binding to full length AP2:cellular effect and molecular model

A Comparison of initiation densities of transient dim clathrin coated structures for parental cell line, isogenic knockout clones 21,31 and 33, FCHO2^{33}-^{WT} and FCHO2^{33_STRING} cell lines.

B Composite structural model of the full length AP2 in complex with FCHO2. The FCHO2 blocks and the AP2 α, β2 and σ subunits are coloured as previously (N1 yellow, N2 orange, N3 red, C block claret, α blue, β2 green and σ2 cyan). The Cμ2 is shown in electrostatic surface representation to demonstrate how the FCHO2 acidic region (acidic residues as red spheres) could interact with the positively charged surface of Cμ2. The length of the linkers separating the various boxes would allow all to bind simultaneously i.e. N1 and N2 binding to Cμ2, N3 binding to β2 and C binding to α-appendage

Zaccai, Kadlecova Figure S10

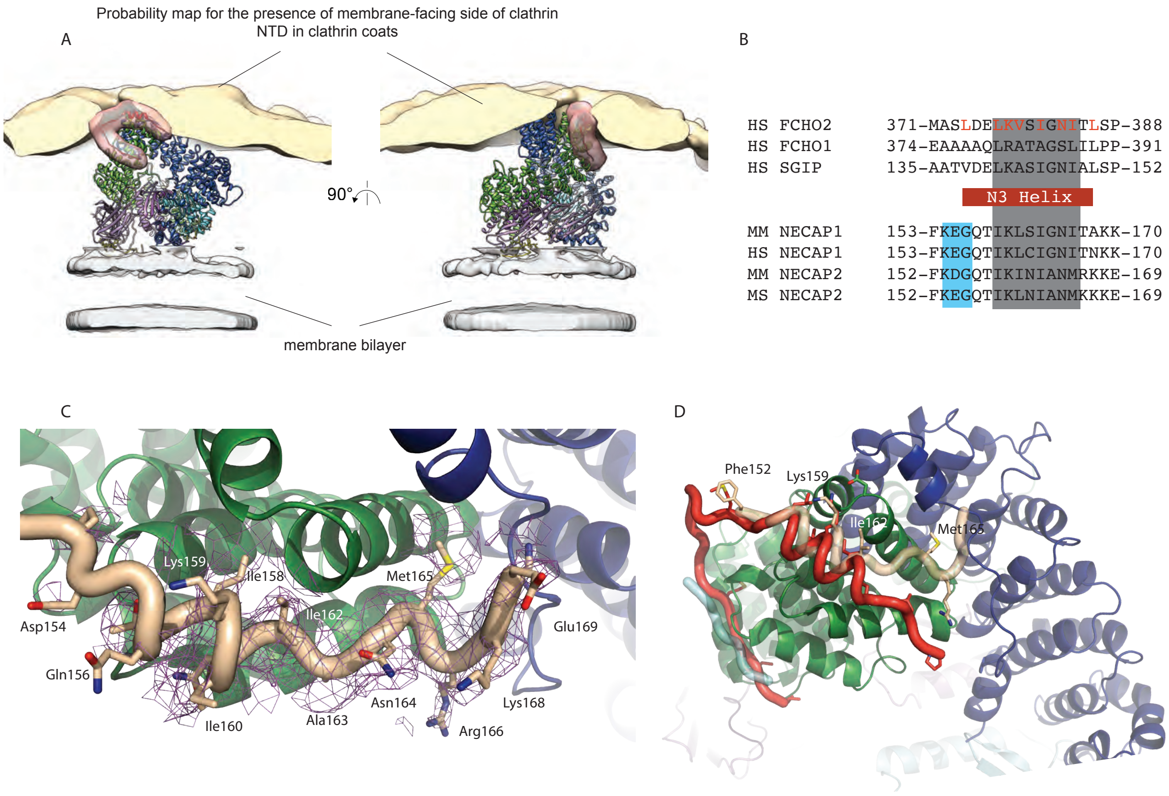

Fig.S10 Competing interactions for FCHO linker blocks on AP2

A The position of the FCHO2 linker relative to AP2 and Clathrin when assembled into a clathrin coat. Membrane-recruited AP2 is depicted as a ribbon model (PDB: 6YAF) and the corresponding EM density is cropped to only illustrate the bilayer region for clarity. The position of the FCHO2 linker was transferred from the X-ray structure of AP2/FCHO2 complex described in this study and is shown as a ribbon model in red and is overplayed with semi-transparent simulated density map derived from cryo EM tomography (*9*). The yellow volume illustrates the probability map of positions of the center of the membrane-proximal face of the clathrin NTDs relative to AP2, at an arbitrarily selected threshold corresponding to approximately 10% of relative subtomogram positions. The FCHO2 linker would be positioned adjacent to and partially overlapping with NTDs of the polymerized clathrin layer and so would be competed off AP2 by clathrin.

B, C, D Possible alternative model interpretation from (*57*) of NECAP2 "Ex" segment in complex with AP2 β2 subunit.

B Sequence homology between N3 helix (376-ELKVSIGNIT-385), mouse NECAP1 "Ex" segments (158-TIKLSIGNIT-167) and mouse NECAP2 "Ex" segment (157-TIKINIANMR-166) allowed NECAP2 "Ex" segment to be positioned in the N3 binding site of the AP2 β2 subunit. Homology is indicated in grey, helical prediction in red and the conserved AP2 binding NECAP1 KEG motif is highlighted in blue).

C Structural comparison between FCHO2 N3 (red) and the alternative NECAP2 "Ex" (yellow) binding position to AP2 β2 (green) and α (blue) subunits built into the clearly helical density

shown in C and refined locally using Coot. The key side chains from NECAP, which are conserved in property to the homologous stretch on N3 (shown in B), are indicated

D In the 3.5Å SPA cryo-EM structure of AP2 in complex with NECAP2 (purple mesh) (*57*), there is α-helical density. The structure of NECAP2 can be readily built into this, on the basis of the FCHO NECAP sequence alignment in B and the density for the NECAP2 specific side chains of Met165 and Arg166 visible. The strongly conserved KEG motif can then be positioned to interact with β2, with the lysine's amine tentatively hydrogen bonding to the main chain carbonyl of β2-Lys494. Importantly, the double mutant KEG to AES abolishes NECAP Ex binding to AP2 (*64*).

If mammalian NECAP 'Ex' segment does indeed occupy the same position as FCHO N3 PAP2•NECAP complex, this would then allow the intervening NECAP main chain to run down the side of the β2 solenoid and would necessitate a repositioning of the KEG sequence originally defined in (*63*) as important for AP2 binding. In such a scenario, FCHO N3 and NECAP Ex would compete for the same binding site on β2 and this will impact their temporal ordering during CCV formation and help to explain their antagonistic actions (*57*). The new positioning of NECAP Ex helix may also have implications for the mechanistic model presented for NECAP function with regards to binding phosphorylated AP2 conformers (*48, 57*). NECAP's integration into CCP will likely be most efficient at the neck/edge of a Ω structure since it will also be connected to SNX9/Amphiphysins BAR domain-containing proteins, which have a preference for binding 'tubular' membrane structures of this curvature (*40*). However, as its Ex domain, like FCHO's N3, sterically overlaps with the clathrin TD layer, NECAP's binding to PAP2 could encourage PAP2 dissociation from the CCP by driving it from the clathrin lattice as well as

probably shifting its equilibrium towards a closed non-membrane-attached form. The net result would be to exclude PAP2 and any attached cargo from the CCP's neck so both removing competition for PtdIns4,5P_2 for SNX9/Amphiphysin-recruited dynamin to bind and to allow complete neck construction, both of which would favour vesicle scission.

Table S1. Cryo-electron tomography data collection.

	AP2	AP2+FCHO
**Microscope, Voltage (keV)**	Titan Krios, 300	
**Detector**	Gatan Quantum K3	
**Energy filter slit width (eV)**	20	
**Electron exposure (e/Å^2) dose fractionation**	~130, uniformly distributed over tilt series	
**Defocus range (µm)**	1.0 – 3.5	
**Tilt scheme (min/max, step)**	-60°/+60, 3°, dose-symmetrical (Hagen scheme)	
**Movie recording**	10 frames per tilt	
**Magnification (times)**	X53,000	
**Pixel size (Å)**	1.701	
**Number of tomograms acquired/used (no.)**	18/17	32/22
**Traced liposomes (no.)**	140	238

Table S2. Subtomogram averaging image processing parameters and statistics.

	AP2	AP2+FCHO
**EMDB**	EMD-????	EMD-????
**EMPIAR**	EMPIAR-??????	EMPIAR-??????
**Particles count (no.):**		
**Initial geometric seeding**	160,263	290,187
**After cross-correlation cleaning in bin8**	112,945	269,280
**After distance cleaning in bin4**	44,514	95,039
**After cross-correlation cleaning in bin4**	41,215	86,311
**After removal of empty classes**	37,600	71,134
**After cross-correlation cleaning in bin2**	27,437	51,868
**EM maps**		
**Symmetry imposed**	none	
**Corrected resolution at FSC threshold 0.143 (Å)**	11.7	9.5

Table S3 Crystallographic data collection and refinement statistics

	AP2 (with β2-FCHO2 chimera)	AP2 in complex with FCHO2linker and TGN	AP2 in complex with SeMetFCHO2 and TGN
**PDB**	7OHO	7OG1	-
**Space group**	P 3_1 2 1	P 2_1	P 2_1
**Cell dimensions (Å, °)** (a, b, c, α, β, γ)	122.0, 122.0, 257.4, 90, 90, 120	92.6, 150.0, 96.4, 90, 112.7, 90	92.0, 146.8, 99.5, 90, 116.4, 90
**Resolution range (Å)** (outer shell)	66.61-2.88 (2.95-2.88)	78.58-3.25 (3.39-3.25)	76.08-3.97 (4.35-3.97)
**R_{merge}**	0.115 (2.019)	0.133 (2.123)	1.109 (4.455)
**R_{pim}**	0.037 (0.649)	0.061 (1.049)	0.458 (1.840)
**Number of observations**	500,376	253,506	276,139
**Number of reflections** (outer shell)	51,118 (3,710)	38,355 (4,690)	20,527 (4,887)
**$\langle(I)/\sigma(I)\rangle$**	13.5 (1.2)	7.2 (0.8)	2.7 (1.0)
**CC(½)**	0.998 (0.529)	0.998 (0.366)	0.956 (0.502)
**Completeness (%)**	99.9 (99.9)	100 (100)	100 (100)
**Multiplicity**	9.8 (9.6)	6.6 (6.0)	6.8 (6.8) (anomalous)
**Wilson B factor ($Å^2$)**	80	102	62
**Resolution (Å)**	61.02 - 2.88	76.54 - 3.25	-
**Reflections** (total / test)	51,118 / 2,547	38,355 / 1,881	
**R / R_{free} (%)**	18.6 / 26.9	20.8 / 31.4	-
**Average B factor ($Å^2$)** (all / AP2 / FCHO / other)	104 / 91 / 176 / 110	145 / 144 / 166 / 177	-
**RMS bond lengths (Å)**	0.008	0.006	-
**RMS bond angles (°)**	1.606	1.497	-
**Ramachandran (%)** (favored / outliers)	87 / 3	90 / 2	

Table S4 Crystallographic data collection and refinement statistics

	**His_6-Cμ2 (apo)**	**His_6-Cμ2-FCHO2** (His6-tagged)	**Cμ2-FCHO2** (cleaved GST)
**PDB**	7OFP	7OHZ	7OI5
**Space group**	P 6_5	P 2_1	C 2
**Cell dimensions (Å, °)** (a, b, c, α, β, γ)	123.8, 123.8, 112.7, 90, 90, 90	55.7, 129.9, 64.4, 90, 102.5, 90	116.0, 55.4, 167.7, 90, 109.0, 90
**Resolution range (Å)** (outer shell)	77.68–1.92 (1.95-1.92)	62.90-2.27 (2.31-2.27)	79.27-2.61 (2.65-2.61)
**Number of observations**	1,321,253	253,807	203,739
**Unique reflections (non-anomalous)**	74,711 (3,642)	40,099 (1,520)	31,089 (1,543)
**Completeness (%)**	99.9 (98.0)	97.1 (72.4)	99.9 (99.3)
**Multiplicity**	17.7 (12.1)	6.3 (4.5)	6.6 (6.2)
**$\langle(I)/\sigma(I)\rangle$**	13.1 (1.0)	7.0 (1.2)	6.6 (1.0)
**R_{merge}**	0.223 (6.379)	0.136 (1.165)	0.163 (1.576)
**R_{pim}**	0.055 (1.912)	0.058 (0.593)	0.068 (0.693)
**CC(½)**	0.999 (0.440)	0.996 (0.602)	0.995 (0.374)
**Wilson B factor (Å^2)**	31	38	52
**Resolution (Å)**	77.68 - 1.92	62.90 - 2.27	79.27 - 2.61
**Reflections** (total / test)	74,663 / 3,819	38,073 / 1,989	30,964 / 1,557
**R / R_{free} (%)**	18.5 / 20.7	27.0 / 31.7	24.5 / 29.4
**Average B factor (Å^2)** (all / AP2 / FCHO / other)	42 / 42 / - / 45	54 / 54 / 65 / 44	73 / 72 / 93 / 53
**RMS bond lengths (Å)**	0.012	0.003	0.003
**RMS bond angles (°)**	1.060	0.643	0.683
**Ramachandran (%)** (favored / outliers)	96 / 1	94 / 0	94 / 0

Table S5 Crystallographic data collection and refinement statistics

	α ear with FCHO1 C block	His$_6$-Cμ2 with FCHO2 C block	His$_6$-Cμ2 with FCHO2 C block
**PDB**	7OHI	7OIT	7OIQ
**Space group**	C 2 2 2_1	P 3_2 2 1	C 2
**Cell dimensions (Å, °)** (a,b,c, α,β,γ)	61.2, 145.5, 88.8, 90, 90, 90	66.4, 66.4, 161.6, 90, 90, 120	118.9, 64.5, 108.4, 90, 112.0, 90
**Resolution range (Å)** (outer shell)	47.60-1.41 (1.43-1.41)	57.47–1.65 (1.68–1.65)	55.70–1.85 (1.88–1.85)
**Number of observations**	873,264	968,797	354,995
**Unique reflections** (outer shell)	73,320 (2,558)	50,587 (2,463)	58,302 (1,599)
**Completeness (%)**	95.9 (67.9)	100.0 (100.0)	89.4 (49.1)
**Multiplicity**	11.9 (6.4)	19.2 (17.0)	6.1 (4.0)
**$\langle (I)/\sigma(I) \rangle$**	17.0 (0.2)	19.0 (1.1)	9.7 (1.1)
**R_{merge}**	0.059 (2.810)	0.082 (2.696)	0.091 (1.064)
**R_{pim}**	0.017 (1.157)	0.019 (0.668)	0.039 (0.577)
**CC(½)**	1.000 (0.253)	1.000 (0.462)	0.995 (0.476)
**Wilson B factor (Å^2)**	23	27	30
**Resolution (Å)**	47.60 - 1.41	57.47 - 1.65	55.70 -1.85
**Reflections** (total / test)	71,365 / 3,608	47,975 / 2,538	58,280 / 2,932
**R / R_{free} (%)**	20.3 / 21.9	19.5 / 21.5	19.5 / 21.7
**Average B factor (Å^2)** (all / AP2 / FCHO / other)	33 / 32 / 55 / 43	35 / 34 / 31 / 44	40 / 40 / 42 / 45
**RMS bond lengths (Å)**	0.006	0.013	0.011
**RMS bond angles (°)**	0.905	1.801	1.658
**Ramachandran (%)** (favored / outliers)	98 / 0	98 / 0	97 / 0

Table S6. Cryo-electron microscopy data collection, refinement and validation statistics for single-particle structures of AP2:β2FCHO2linker chimaera in solution.

**Microscope and Detector**	**Titan Krios, K3**	
**Automated data collection software**	Thermo Scientific EPU	
**Voltage (kV)**	300	
**Energy filter slit width (eV)**	20	
**Electron exposure (e/Å^2)**	47.3	
**Exposure rate (e-/pixel/sec)**	15.3	
**Defocus range (μm)**	0.8-2.8	
**Movie recording**	48 frames	
**Magnification (nominal)**	130,000	
**Pixel size (Å)**	0.326	
**Number of micrographs**	13,250	
**Initial number of particles / number used for refinement**	718,511 / 452,076	
	**Consensus**	**Cμ2-in N1+N2**
**EMDB/PDB ID**	XXXX/XXXX	XXXX/XXXX
**Final number of particles (no.)**	143,815	28,392
**Map resolution, FSC masked (0.143/0.500) (Å)**	3.8 / 4.3	6.2/7.2
**Map resolution, FSC unmasked (0.143/0.500) (Å)**	3.8/4.4	6.3/7.3
**Resolution map vs model FSC (0.500) (Å)**	3.9	
**Local resolution range (Å)**	3.5-5.0	5.6-14.0
**Sphericity (3DFSC)**	0.87	0.94
**B factor for sharpening**	-162	-206
**Initial model, PDB ID**	2vgl	
**Atomic modelling refinement**	Phenix.real_space_refine	
**Non-hydrogen atoms**	13,707	
**Protein residues**	1717	
**Ligands**	0	
**CCvolume**	0.80	
**CCmask**	0.78	
**ADP (B-factors)**		
**Iso/Aniso (#)**	13707/0	
**Protein (min/max/mean)**	12.41/123.92/55.57	
**Ligand**	---	
**R.m.s. deviations**		
**Bond length (Å)**	0.003	
**Bond angle (°)**	0.549	
**C-beta**	0.00	
**Validation**		
**Molprobity score**	1.74	
**Clashscore**	9.53	
**Poor rotamers (%)**	0	
**CaBLAM outliers (%)**	2.07	

**Ramachandran plot**	
**Favoured (%)**	96.42
**Allowed (%)**	3.58
**Outliers (%)**	0.0

Supplementary Videos

Video S1 Super-resolution live-cell imaging of CME dynamics by eTIRF-SIM in U-2 OS cells expressing m-Scarlet-FCHO2^{WT} and eGFP-CLCa. Movies were acquired in sequential mode for 6 minutes with 2s frame intervals.

Upper panel: Averaged TIRF time-lapse movie: For each channel the nine frames of raw TIRF–SIM data were gathered with three different phases and angles at every time point and then averaged for CCP detection and tracking with CMEanalysis analysis suite and custom Matlab scripts. The resulting detected spatial coordinates of valid tracks were superimposed on the final eTIRF-SIM reconstructed time-lapse movie (Lower panel).

Video S2 Detail of eTIRF-SIM reconstructed time-lapse movie capturing eGFP-CLCa and mScarlet-FCHO2^{WT} in U-2 OS cells.

Video S3 Formation of a single CCP imaged by eTIRF-SIM for 65 seconds with 2s frame interval**.** Initiation, growth, and scission of visualized with eGFP-CLCa.

Video S4 Conformational variability in single particle EM reconstruction of AP2 chimera.

3D variability analysis (3DVA; CryoSPARC) was carried out on the majority dataset of AP2 FCHO2 chimera in which the stacked α-solenoid structures of AP2 contract with a concurrent elongation of the long axis of the AP2 core particle. This motion may be a step towards opening of the AP2 bowl, wherein Cμ2 is ejected and the α and β2 trunks coverage by a distance of ~16Å
